# Inhibition of dipeptidyl peptidase-4 ameliorates cardiac ischemia and systolic dysfunction by up-regulating the FGF-2/EGR-1 pathway

**DOI:** 10.1371/journal.pone.0182422

**Published:** 2017-08-03

**Authors:** Masayoshi Suda, Ippei Shimizu, Yohko Yoshida, Yuka Hayashi, Ryutaro Ikegami, Goro Katsuumi, Takayuki Wakasugi, Yutaka Yoshida, Shujiro Okuda, Tomoyoshi Soga, Tohru Minamino

**Affiliations:** 1 Department of Cardiovascular Biology and Medicine, Niigata University Graduate School of Medical and Dental Sciences, Niigata, Japan; 2 Division of Molecular Aging and Cell Biology, Niigata University Graduate School of Medical and Dental Sciences, Niigata, Japan; 3 Department of Structural Pathology, Institute of Nephrology, Graduate School of Medical and Dental Sciences, Niigata University Graduate School of Medical and Dental Sciences, Niigata, Japan; 4 Division of Bioinformatics, Niigata University Graduate School of Medical and Dental Sciences, Niigata, Japan; 5 Institute for Advanced Biosciences, Keio University, Yamagata, Japan; Rutgers New Jersey Medical School, UNITED STATES

## Abstract

Dipeptidyl peptidase 4 inhibitors are used worldwide in the management of diabetes, but their role in the prevention or treatment of cardiovascular disorders has yet to be defined. We found that linagliptin, a DPP-4 inhibitor, suppressed capillary rarefaction in the hearts of mice with dietary obesity. Metabolomic analysis performed with capillary electrophoresis/mass spectrometry (LC-MS/MS) showed that linagliptin promoted favorable metabolic remodeling in cardiac tissue, which was characterized by high levels of citrulline and creatine. DNA microarray analysis revealed that the cardiac tissue level of early growth response protein 1 (EGR-1), which activates angiogenesis, was significantly reduced in untreated mice with dietary obesity, while this decrease was inhibited by administration of linagliptin. Mature fibroblast growth factor 2 (FGF-2) has a putative truncation site for DPP-4 at the NH2-terminal, and LC-MS/MS showed that recombinant DPP-4 protein cleaved the NH2-terminal dipeptides of mature FGF-2. Incubation of cultured neonatal rat cardiomyocytes with FGF-2 increased Egr1 expression, while it was suppressed by recombinant DPP-4 protein. Furthermore, vascular endothelial growth factor-A had a critical role in mediating FGF-2/EGR-1 signaling. In conclusion, pharmacological inhibition of DPP-4 suppressed capillary rarefaction and contributed to favorable remodeling of cardiac metabolism in mice with dietary obesity.

## Introduction

Dipeptidyl peptidase 4 (DPP-4; also known as CD26) is a glycoprotein that regulates a broad spectrum of biological processes through catalytic and/or non-catalytic actions [1, 2]. It is well known that DPP-4 promotes the pathology of diabetes via catalytic degradation of glucagon-like peptide-1 (GLP-1) and gastric inhibitory peptide (GIP), which induce postprandial secretion of insulin by pancreatic β-cells. In addition to promoting insulin secretion, GLP-1 and GIP are involved in the production of nitric oxide (NO) and atrial natriuretic peptide (ANP)[2]. As well as these molecules with a critical role in the maintenance of cardiovascular homeostasis, many other biologically important mediators have putative truncation sites for DPP-4, and it is quite possible that DPP-4 is crucial for down-regulating molecules with a beneficial (and sometimes detrimental) role in cardio-metabolic disorders [3, 4]. The circulating level of DPP-4 is correlated with visceral adiposity, inflammation, and the glycated hemoglobin (HbA1c) level, and pharmacological suppression of DPP-4 contributes to reduction of HbA1c mainly via GLP-1- and GIP-mediated pathways [2, 5, 6]. Accordingly, DPP-4 inhibitors have become one of the first-line treatments for diabetes. Heart failure is also associated with elevated circulating DPP-4 levels, and DPP-4 activity increases with the progression of heart failure [7, 8]. It is well known that systemic insulin resistance is correlated with development of heart failure, while heart failure per se provokes systemic glucose intolerance [9–11]. There is evidence of a close pathological interaction between systemic metabolic disorders and heart failure [12, 13]. It is well known that metabolic remodeling occurs in the failing heart, leading to impaired cardiac energetics and eventually to structural remodeling [14, 15]. Therefore, development of therapies targeting cardiac metabolism could be crucial for combating heart failure[16]. There is controversy as to whether DPP-4 inhibitor therapy contributes to suppression of pathologic changes in heart failure. In animal models of diabetic cardiomyopathy or left ventricular pressure overload, pharmacological or genetic suppression of DPP-4 generally ameliorates systolic and/or diastolic cardiac dysfunction [17–20]. Despite the results of animal studies suggesting that DPP-4 inhibitors are promising candidates for treatment of heart failure, human studies have not shown a beneficial effect so far [21]. In some studies, DPP-4 inhibitor therapy slightly increased the risk of heart failure [22, 23], while other studies (including recent large-scale clinical trials) have found no additional risk [24–26]. These conflicting results promoted us to characterize cardiac metabolism in the diabetic state with or without DPP-4 inhibitor treatment. In a murine model of left ventricular pressure overload, capillary rarefaction develops in the left ventricle and this has been shown to contribute to progression of pathologic changes in the failing heart [27]. Metabolites are delivered to the tissues by capillaries, so in addition to characterizing the cardiac metabolic profile, we evaluated capillary network formation in cardiac tissue under metabolic stress with or without DPP-4 inhibitor treatment. In a murine model of dietary obesity, we found that a DPP-4 inhibitor (linagliptin) suppresses capillary rarefaction in cardiac tissue and promotes favorable metabolic remodeling, characterized by elevated levels of citrulline and creatine.

## Materials and methods

### Animal models

These animal experiments were conducted in compliance with the protocol reviewed and approved by the Institutional Animal Care and Use Committee of Niigata University and approved by the President of Niigata University. C57BL/6NCr male mice were purchased from SLC Japan (Shizuoka, Japan). Mice were kept under standard housing temperature (23–24°C) in a 12hr light-dark cycle. Four-week-old mice were fed a high-fat diet (HFD32, CLEA Japan) or normal chow for 8 weeks, and were analyzed at 12 weeks of age. The DPP-4 inhibitor linagliptin was supplied by Boehringer Ingelheim, and was added to the drinking water (10 mg/kg/day) for 8 weeks from 4 weeks of age. All mice were randomized to normal chow and HFD group with or without the DPP-4 inhibitor. All the experiments were done under blinded condition. For the oral glucose tolerance test, mice were fasted for 6 hours from 9 am and glucose was given orally at a dose of 1g/kg. For the insulin tolerance test, mice were given 1U/kg insulin intraperitoneally. Blood glucose levels were measured with a glucose analyzer (Roche Diagnostics) at 0, 15, 30, 60, and 120 min after glucose or insulin loading. Systolic blood pressure was measured by the tail-cuff method with an indirect sphygmomanometer (Softron BP-98A). After the animals were euthanized by intraperitoneal barbiturate injection, tissues were quickly corrected for further analyses. All animal experiments followed ARRIVE guidelines (S1 File).

### Histological and physiological analyses

Cardiac tissue samples were harvested, fixed overnight in 10% formalin, embedded in paraffin, and sectioned for immunohistochemistry. Capillaries were stained with isolectin GS-IB4 from Griffonia simplicifolia biotin-XX conjugate. Cell membranes of cardiomyocytes were stained with a Wheat Germ Agglutinin Alexa FluorR 488 conjugate (1:10, Life Technologies, W11261) to allow calculation of the cross-sectional area (CSA). The number of capillaries and CSA were determined in four randomly selected fields at x400 magnification per sample. Capillary density was estimated as follows: density = number of capillaries/number of cardiomyocytes /CSA. Tissue hypoxia was assessed with Hypoxyprobe-1 (Hypoxyprobe, Inc. HP1-100) according to the manufacturer’s instructions. Briefly, intraperitoneal injection of pimonidazole (60 mg/kg) was performed 60 minutes before harvesting hearts, which were fixed in 10% formalin overnight. Then cardiac tissue specimens were embedded in paraffin, cut into sections 5 μm thick, and stained with Hypoxyprobe-1 monoclonal antibody (NPI, Inc. Burlington, clone 4.3.11.31), which binds to protein adducts of pimonidazole in hypoxic regions. Stained sections were photographed with a Biorevo (Keyence, Japan). Echocardiography was performed under isoflurane anesthesia (1–3%) with a Vevo770 High Resolution Imaging System (Visual Sonics Inc., Canada).

### DPP-4 activity assay

Plasma samples were collected from mice at 12 weeks of age and 20 μl of plasma was used to measure DPP-4 activity with a DPPIV/CD26 Assay Kit for Biological Samples (Enzo Life Science) according to the manufacturer’s instructions.

### Metabolome analyses

Metabolomic analysis was performed by Soga et al. using capillary electrophoresis/ mass spectrometry as described previously [28].

### Tissue total Nitric Oxide assay

Cardiac tissues (50 mg) were homogenized with assay buffer and deproteinized with 4 M perchloric acid and 2 M potassium hydroxide. Then the supernatant was used for measurement of nitric oxide with a Nitric Oxide Assay Kit (Abcam; ab65328) according to the manufacturer’s instructions.

### RNA analysis

Total RNA (1 μg) was isolated from tissue samples with RNA-Bee (TEL-TEST INC.). Quantitative PCR was performed by using a Light Cycler 480 (Roche) with a Taqman Universal Probe Library and Light Cycler 480 Probes Master (Roche) according to the manufacturer’s instructions. Vegfa164 was analyzed with Light Cycler 480 SYBR Green I Master (Roche) according to the manufacturer’s instructions. The following primers were used.

Mouse primers:

*Ass1*; 5’-TGTGCTTATAACCTGGGATGG-3’, 5’-ACAGTGCAGTGAACCACTCG-3’.

*Asl*; 5’-ACATGGCCTCGGAGAGTG-3’, 5’-ATGGACGCGTTGAACTTCTC-3’.

*Arg1*; 5’-CAAGGTGGCAGAAGTCAAGA-3’, 5’-GCTTCCAATTGCCAAACTGT-3’.

*Arg2*; 5’-AGCAGAGCTGTGTCAGATGG-3’, 5’-GGCATGGCCACTAATGGTA-3’.

*Otc*; 5’-CAGCGAAATTCGGAATGC-3’, 5’-CTAGCATCCGGCTCATAACC-3’.

*Gatm*; 5’-GGTGCACTACATCGGCTCTC-3’, 5’-CAGGAATTTCGGGAGGAAG-3’.

*Gamt*; 5’-TGGGAGACCCCCTATATGC-3’, 5’-CGAAGCCCACTTCCAAGAC-3’.

*Egr1*; 5’-CCTATGAGCACCTGACCACA-3’, 5’-TCGTTTGGCTGGGATAACTC-3’.

*Nos3*; 5’-Ccagtgccctgcttcatc-3’, 5’-gcagggcaagttaggatcag-3’

*Rplp0*; 5’-GATGCCCAGGGAAGACAG-3’, 5’-ACAATGAAGCATTTTGGATAATCA-3’.

Rat primers:

*Vegfa164*; 5’-CAGAAAATCACTGTGAGCCTTGTT-3’, 5’-CTTTCCGGTGAGAGGTCTGC-3’.

*Egr1*; 5’-CGAACAACCCTACGAGCAC-3’, 5’-GCGCCTTCTCGTTATTCAGA-3’.

*Rplp0*; 5’-GATGCCCAGGGAAGACAG-3’, 5’-CACAATGAAGCATTTTGGGTAG-3’.

Rplp0 (mouse) or Rplp0 (rat) was used as the internal control for all studies.

### Microarray analysis

Total RNA was isolated from tissue samples with an RNeasy Microarray Tissue Mini Kit (Qiagen). Microarray analysis was performed by the Chemicals Evaluation and Research Institute (CERI), Japan. The gene expression data obtained were deposited in the Gene Expression Omnibus database (GSE98226).

### Trypsin digestion for mass spectrometric analysis

After incubation of rFGF2 with or without rDPP4, the reaction mixture (40 μl) was transferred into a new microcentrifuge tube for “tube gel digestion”, a modified in-gel digestion procedure [29], to generate dithiothreitol-reduced iodoacetamide-alkylated tryptic peptides for nano-flow liquid chromatography coupled with tandem mass spectrometry (LC-MS/MS). Briefly, the reaction mixture was mixed with 15 μl of acrylamide (40% T, acrylamide: N, N’-methylene bis(acrylamide) at 9:1 by weight) and 0.5 μl each of 10% ammonium persulfate and N, N, N’, N’-tetramethylenethylenediamine, followed by polymerization for 60 min at room temperature. The polymerized tube gel was immersed in 50% methanol with 7% acetic acid for 30 min, and then was kept at 4°C in 5% acetic acid. Tube gels were subjected to in-gel digestion with trypsin (Sigma, Proteomics sequencing grade), essentially by the method of Katayama et al. [30].

### Mass spectrometry

Peptides generated from each sample were dissolved in 15 μl of 0.3% formic acid, and aliquots (3 μl) were injected into a nano-flow liquid chromatograph (Eksigent nanlLC 415 with ekspert cHiPLC, Sciex) coupled with a tandem mass spectrometer (TripleTOF5600+, Sciex) via a nano ESI ion source. Analysis of each sample was conducted in duplicate in the trap and elute modes using a ChromeXP C18 Chip column (200 μm × 0.5 mm) as the trap column and a ChromeXP C18 Chip column (75 μm × 150 mm) as the analytical column. Mobile phases A and B were 0.1% formic acid and 0.1% formic acid in acetonitrile, respectively. Peptides were eluted with a gradient from 2% to 32% of mobile phase B over 20 min at 300 nl/min. The MS spectrum (250 msec) and 10 MS/MS spectra (100 msec each) were acquired in the data-dependent mode. The dynamic exclusion time was set at 12 sec. Auto-calibration using 50 fmol of bovine serum albumin tryptic peptides (KYA Technology, Tokyo, Japan) was performed after assay of every 3 samples.

### Protein identification

Raw data generated by Analyst TF1.6 (Build 6211) were converted to mascot generic files by MS Converter (Sciex). The two mascot files generated from duplicate runs for each sample were merged, and searched against UniProt human reference database (released May 2015) under the ESI-QUAD-TOF instrumental setting. Peptide and MS/MS tolerances were set at ±20 ppm and ±0.1 Da, respectively. Modification settings were as follows: carbamidomethylation of cysteine was the fixed modification and the variable modifications were deamidation of asparagine and/or glutamine, N-terminal glutamine to pyroglutamate, and N-terminal glutamate to pyroglutamate with oxidation of methionine. A maximum of two missed cleavages was allowed. The target false discovery rate was < 1%.

### Cell culture

Rat neonatal ventricular cardiomyocytes (NRVMs) were prepared from the hearts of 2- to 3-day-old Wistar rats as described previously [27]. Animals were sacrificed by decapitation and cardiac tissues were quickly corrected for further processes. In this assay, atriums and vessels were removed and ventricles were digested for further procedures. After the digestion of cardiac tissues, all cells were incubated for 60min, then the supernatant (NRVMs) and the attached cells (Fibroblasts) were further processed to culture these cells respectively. The cells were incubated at 37°C in a 5% CO2 atmosphere and the medium was changed after 24 hr. In some studies, cells were incubated with recombinant human basic fibroblast growth factor at 35 ng/ml (hFGF basic/FGF2, Cell Signaling Technology, #8910LF) with or without 0.1 μg/ml of recombinant human DPPIV/CD26 (R&D 1180-SE) at 37°C in an incubator. Human umbilical vein endothelial cells (HUVECs) were cultured according to the manufacturer’s instructions (Lonza).

### Tube formation assay

Cells were incubated with 35 ng/ml of recombinant human basic fibroblast growth factor (hFGF basic/FGF2, Cell Signaling Technology, #8910LF) with or without 0.1 μg/ml of recombinant human DPPIV/CD26 (R&D 1180-SE) for 1 hr at 37°C in an incubator. Then the tube-forming assay (Cell Biolabs, Inc.) was performed according to the manufacturer’s instructions. Briefly, HUVECs were seeded in 96-well plates coated with ECM gel and were incubated in EGM-2 medium (Lonza) without growth factors (hEGF, hFGF-B, VEGF, and R3-IGF-1), or with reduced serum (0.1% FBS) and recombinant FGF2 with or without recombinant DPP4 protein. After incubation for 12 h at 37°C, microscopy was performed and the tube length was quantified by using the Angiogenesis Analyzer plug-in and ImageJ software (NIH).

### RNA interference

Small interfering RNA (siRNA) targeting Egr1 [a mixture of si-Egr1 (RSS332331, RSS332332, and RSS332333) (total 50 pmol)] and the corresponding negative control (45–2001) (50 pmol) were purchased from Invitrogen. Cells were transfected with these siRNAs by using Lipofectamine RNAi MAX (Invitrogen, #13778–150) and Opti-MEM (Gibco by Life Technologies, #31985–062). The medium was changed at 24 hours after addition of the siRNAs, and the cells were incubated for another 48 hours before studies were performed.

### Enrichment analysis

Genes in transcriptome analysis and metabolites in metabolome analysis were mapped to the identifiers defined in the KEGG (Kyoto Encyclopedia of Genes and Genomes) database (downloaded on May 21, 2014). The number of genes and metabolites in each category of KEGG with more than a cutoff was counted. Whole genes in mouse genome and whole metabolites estimated to be present from the genome were used as the reference sets in the enrichment analyses for transcriptomics and metabolomics analysis, respectively. If a gene is mapped to an enzyme reaction defined in KEGG, the substrate and product compounds were supposed as present in the species, in order to estimate the number of whole metabolites to be present. Subsequently, significantly enriched KEGG categories in both transcriptomics and metabolomics analyses were extracted based on q-values [31] from the fisher’s exact test performed by using R (http://www.r-project.org/).

### Statistical analysis

Statistical analysis was done with SPSS version 20. Data are shown as the mean ± SEM. Differences between groups were examined by the two-tailed Student’s t-test, or by two-way ANOVA followed by Tukey’s multiple comparison test for comparisons among more than two groups. For all analyses, P<0.05 was considered statistically significant.

## Results

### Obesity-induced capillary rarefaction is suppressed by inhibition of DPP-4

**Fig 1:**
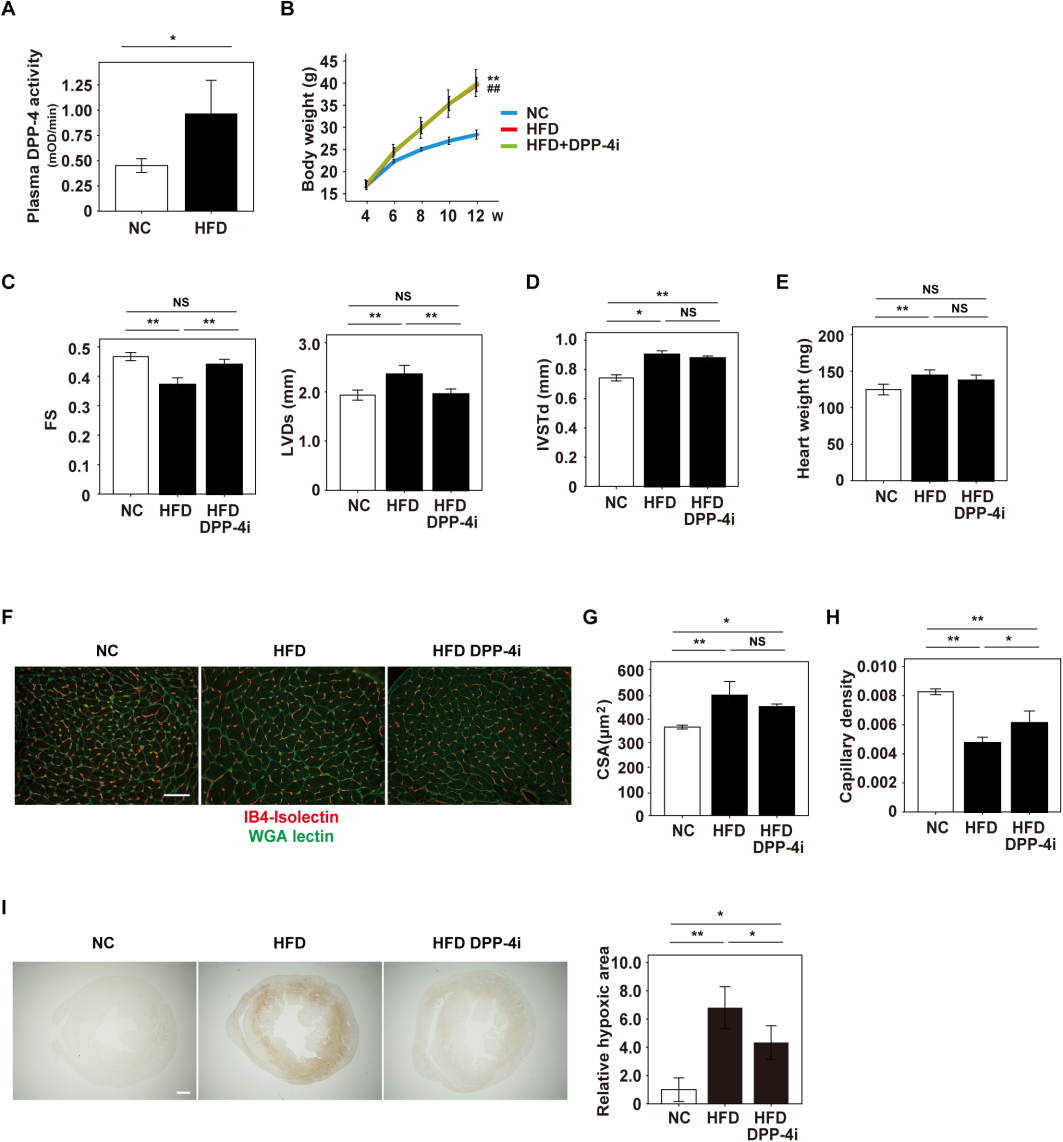
Linagliptin ameliorates capillary rarefaction in cardiac tissue due to metabolic stress. (A) Plasma DPP-4 activity of mice fed normal chow (NC) or a high fat diet (HFD) (n = 3,3). (B) Body weight of mice fed NC, HFD, or HFD+linagliptin, a DPP-4 inhibitor (HFD+DPP-4i) (n = 8,9,7). (C-I) Cardiac features were analyzed in the indicated groups: echocardiography (C,D) [fractional shortening (FS), left ventricular systolic dimension (LVDs) (n = 10,14,19), interventricular septal thickness at end-diastole (IVSTd) (n = 13,18,19)], heart weight (E) (n = 13,14,14), IB4-isolectin and WGA staining of cardiac tissue (F) (scale bar = 100 μm), cross-sectional area (CSA) (G) (n = 3,3,3), capillary density (number of microvessels/number of cardiomyocytes/CSA) (H) (n = 3,3,3), and hypoxic probe staining (I) (scale bar = 500 μm). Right panel in (I) shows quantification of relative hypoxic area (n = 3,6,5). Data were analyzed by the 2-tailed Student’s t-test (A), or 2-way ANOVA followed by Tukey’s multiple comparison test (B,C,D,E,G,H and I). *P<0.05, **P<0.01. All values represent the mean ± s.e.m. NS = not significant.

We generated a murine model of dietary obesity by feeding C57BL/6 mice a high-fat diet (HFD). Plasma dipeptidyl peptidase-4 (DPP-4) activity was significantly increased in these mice (Fig 1A). Metabolic stress is known to promote pathologic changes in cardiac tissue [32], so we investigated whether pharmacological inhibition of DPP-4 could suppress the development of cardiac dysfunction. HFD mice showed weight gain and administration of linagliptin (a DPP-4 inhibitor) did not result in a leaner phenotype, although glucose intolerance was improved (Fig 1B, S1 Fig). Insulin tolerance test indicated no change in the systemic insulin sensitivity (S1A Fig). Systolic and diastolic blood pressures, and food intake were similar in the HFD and HFD+DPP4i mice (S1B and S1C Fig). Systolic cardiac dysfunction and left ventricular dilatation developed in HFD mice, and these changes were suppressed by linagliptin treatment (Fig 1C). HFD mice showed cardiac hypertrophy as well as cardiomyocyte hypertrophy, but these changes were not ameliorated by linagliptin (Fig 1D–1G). In a murine model of left ventricular pressure overload, capillary rarefaction was reported to develop in the decompensated phase of heart failure and contributed to progression of this condition [12, 27]. We found that dietary obesity led to capillary rarefaction and hypoxia in the cardiac tissue of HFD mice, while linagliptin treatment ameliorated these changes (Fig 1F–1I). These results indicated that pharmacological suppression of DPP-4 activity prevented cardiac dysfunction by inhibiting the development of capillary rarefaction in cardiac tissue due to metabolic stress.

### DPP-4 inhibition leads to favorable metabolic remodeling in the cardiac tissue of a murine obese model

**Fig 2:**
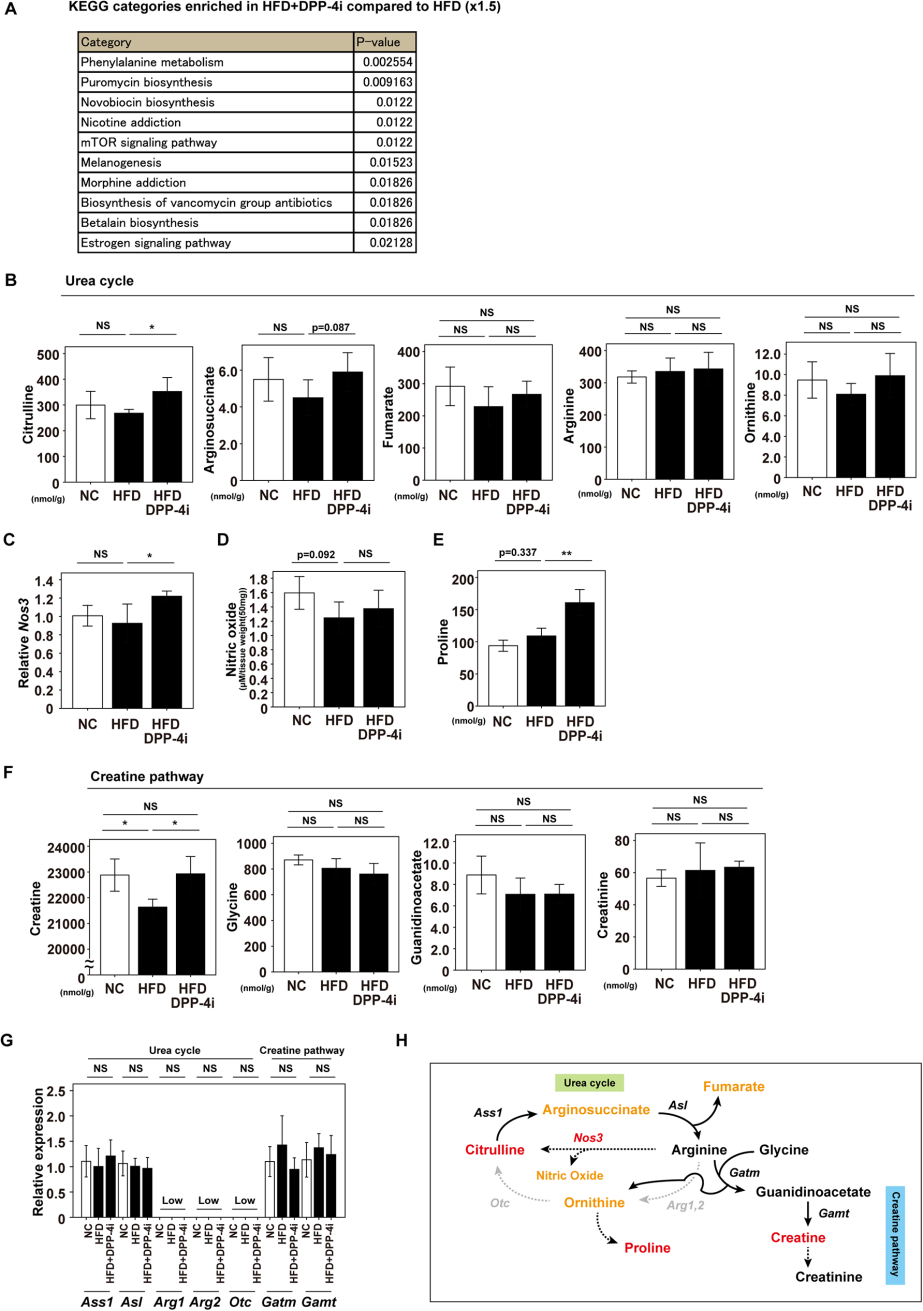
Characterization of the cardiac urea cycle and creatine pathway. (A) Metabolomic data on pathway enrichment analysis in cardiac tissue. KEGG categories enriched by 1.5-fold in mice on a high fat diet (HFD) receiving linagliptin, a DPP-4 inhibitor (HFD+DPP-4i) (n = 5), compared to untreated HFD mice are described (n = 5). (B, E, F) Metabolites of the urea cycle (B, E) or creatine pathway (F) were assessed in cardiac tissue by metabolomic analysis with capillary electrophoresis/ mass spectrometry (CE/MS) in mice fed normal chow (NC), HFD, or HFD+DPP-4i (n = 5,5,5). (C, D) Quantitative PCR study for *Nos3* (C)(n = 6,6,7) and nitric oxide level (D)(n = 8,8,5) in the cardiac tissues of indicated mice groups (n = 6,6,7). (G) Quantitative PCR study for enzymes related to urea cycle (*Ass1*, *Asl*, *Arg1*, *Arg2* and *Otc*) or creatine pathway (*Gatm* and *Gamt*) (n = 11,11,13). The description “Low” indicates CT value to be mostly over 35 or undetected. (H) Scheme summarizing the findings of metabolomic analysis and quantitative PCR. Metabolites or transcripts are displayed by using the following colors: red (significantly increased in HFD mice receiving linagliptin compared to untreated HFD mice), orange (increased in HFD mice receiving linagliptin, but not significantly), black (no change), and gray (not detected or low). Data were analyzed by 2-tailed Student’s *t*-test (A,G), with 2-way ANOVA followed by Tukey’s multiple comparison (B–F) except for arginosuccinate that was analyzed by Student’s *t*-test. *P<0.05, **P<0.01. All values represent the mean ± s.e.m. NS = not significant.

It is well known that energy consumption is high in cardiac tissue. ATP is mainly generated from fatty acids through oxidative phosphorylation under physiological conditions, and it is generally accepted that glucose utilization increases with progression of heart failure. Metabolic remodeling is considered to underlie the progression of heart failure [14, 16], so we investigated the cardiac metabolic profile of HFD mice with or without linagliptin treatment. Metabolomic analyses were done with capillary electrophoresis/mass spectrometry (S1 and S2 Tables). We found that administration of linagliptin to HFD mice led to enrichment in KEGG categories including “Phenylalanine metabolism” and “mTOR signaling pathway” (Fig 2A). To identify the specific pathways involved, we focused on metabolites or metabolic pathways in cardiac tissue that showed differences between untreated HFD mice and HFD mice receiving linagliptin. Compared to untreated HFD mice, citrulline showed a significant increase in the cardiac tissue of HFD mice treated with linagliptin (Fig 2B). Interestingly, in addition to products of citrulline metabolism such as arginosuccinate, there was an increase of metabolites related to the urea cycle such as fumarate and ornithine (Fig 2B). The arginine level was similar in both groups of mice (Fig 2B), but a transcript for nitric oxide synthase, endothelial (Nos3), was significantly higher in HFD mice receiving linagliptin (Fig 2C), and nitric oxide was also increased in this group (Fig 2D). Furthermore, proline was significantly higher in HFD mice receiving linagliptin, but further studies are needed to determine the biological implications (Fig 2E). We also found that the cardiac tissue level of creatine was significantly increased in HFD mice treated with linagliptin (Fig 2F), while the levels of other metabolites such as glycine, guanidinoacetate, and creatinine were similar in the two groups (Fig 2F). Next, we analyzed the cardiac tissue levels of transcripts for enzymes involved in the urea cycle and creatine pathway, finding that some enzymes were expressed prominently (Ass1, Asl, Gatm, and Gamt) and others were expressed at low levels (Arg1, Arg2, and Otc) (Fig 2G). These results suggested that pathways such as the urea cycle and creatine pathway were partly activated by pharmacological suppression of DPP-4, contributing to favorable metabolic remodeling in cardiac tissue that was characterized by elevation of citrulline and creatine (Fig 2H). ATP levels were similar between untreated HFD mice and HFD mice receiving linagliptin (S2A Fig). Tyrosine, methionine, and tryptophan were higher in HFD mice receiving linagliptin, while norepinephrine and glutamate levels were similar in both groups (S2B and S2C Fig). Compared to untreated HFD mice, HFD mice receiving linagliptin showed a negative enrichment in KEGG categories including “Citrate cycle” and “Glyoxylate and dicarboxylate metabolism”, while UDP-glucuronate was significantly reduced in the linagliptin treatment group (S2D and S2E Fig). Further studies are needed to elucidate the implications of these changes of the cardiac tissue metabolic profile induced by linagliptin in HFD mice.

### Suppression of DPP-4 increases early growth response protein 1 expression

**Fig 3:**
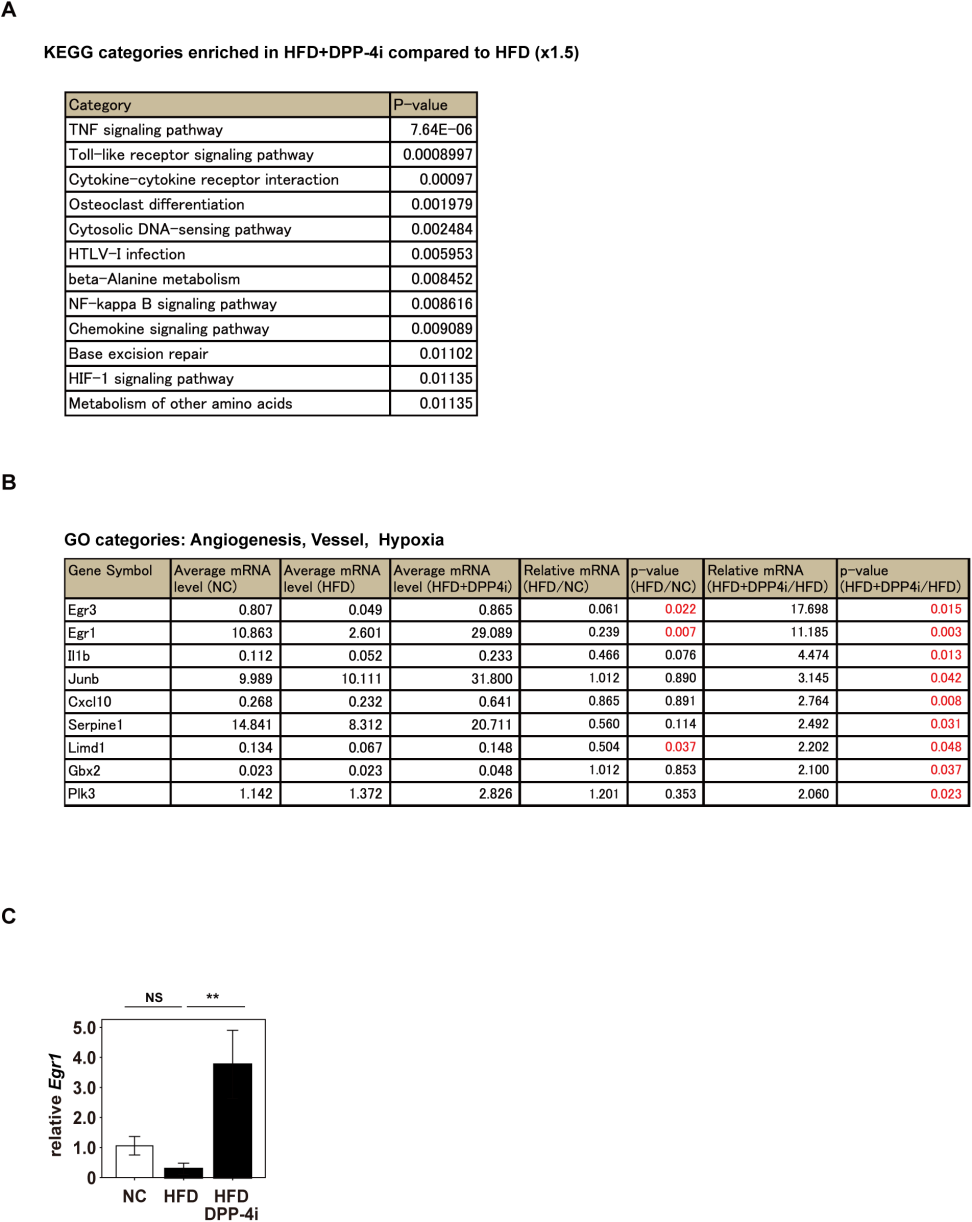
Linagliptin increases Egr1 in cardiac tissue. (A) DNA microarray analysis data for cardiac tissues. KEGG categories enriched by 1.5-fold in mice on a high fat diet (HFD) receiving linagliptin, a DPP-4 inhibitor (HFD+DPP-4i) (n = 3), compared to untreated HFD mice (n = 3) are described. (B) In DNA microarray analysis, transcripts with GO terms such as “Angiogenesis”, “Vessel” or “Hypoxia” were extracted. The top 9 molecules showing higher expression in HFD+DPP-4i mice compared to untreated HFD mice are displayed [normal chow (NC) (n = 3), HFD (n = 3) and HFD+DPP-4i(n = 3)]. (C) Quantitative PCR for *Egr1* in cardiac tissues of the indicated mice (n = 6,6,6). Data were analyzed by the 2-tailed Student’s t-test (A, B), or 2-way ANOVA followed by Tukey’s multiple comparison test (C). *P<0.05, **P<0.01. All values represent the mean ± s.e.m. NS = not significant.

Since pharmacological inhibition of DPP-4 contributed to suppression of capillary rarefaction in the cardiac tissue of HFD mice (Fig 1H), we investigated the underlying mechanisms further. When DNA microarray analysis was performed, the HFD mice receiving linagliptin showed the enrichment in KEGG categories including “HIF-1 (Hypoxia inducible factor-1) signaling pathway” compared to untreated HFD mice (Fig 3A). Among several molecules showing a significant increase in the HFD mice receiving linagliptin compared to the untreated HFD mice, we focused on molecules with terms for GO categories such as “Angiogenesis”, “Vessel” or “Hypoxia”. Among them, early growth response protein 1 (EGR-1) was predominantly expressed in the mice receiving linagliptin (Fig 3B). DNA microarray data suggested that Egr1 expression in cardiac tissue was reduced by the HFD, while DPP-4 inhibitor treatment resulted in a significant increase (Fig 3B). We confirmed that Egr1 expression was significantly higher in HFD mice receiving linagliptin compared to untreated HFD mice by performing quantitative PCR (Fig 3C). EGR-1 is reported to promote angiogenesis [33]. Capillary rarefaction developed in the cardiac tissue of mice with dietary obesity, while linagliptin treatment led to an increase of capillary density (Fig 1F and 1H). Taken together, our data suggested that DPP-4 inhibitors may suppress cardiac remodeling by increasing angiogenesis in this organ through induction of the pro-angiogenic transcription factor EGR-1. Interestingly, there were transcripts with GO terms such as “Angiogenesis”, “Vessel” or “Hypoxia” that were shown to increase or reduce in the HFD+DPP-4 inhibitor group compared to the HFD group, indicating that several other pathways involved in pro- or anti-angiogenesis would be regulated by DPP-4 (Fig 3B, S3 Fig, S2 File). We found that FGF-2 also increased *Vegfa* expression in fibroblasts, and this increase was suppressed by DPP-4 treatment (S4A Fig). Genetic depletion of *Egr1* in fibroblasts resulted in the reduction of *Vegfa* in these cells, indicating that DPP-4 may also induce capillary rarefaction in cardiac tissues by the suppression of FGF-2/EGR1-mediated angiogenic response in fibroblasts (S4B Fig).

### DPP-4 cleaves NH2-terminal dipeptides of fibroblast growth factor 2 and suppresses angiogenesis

**Fig 4:**
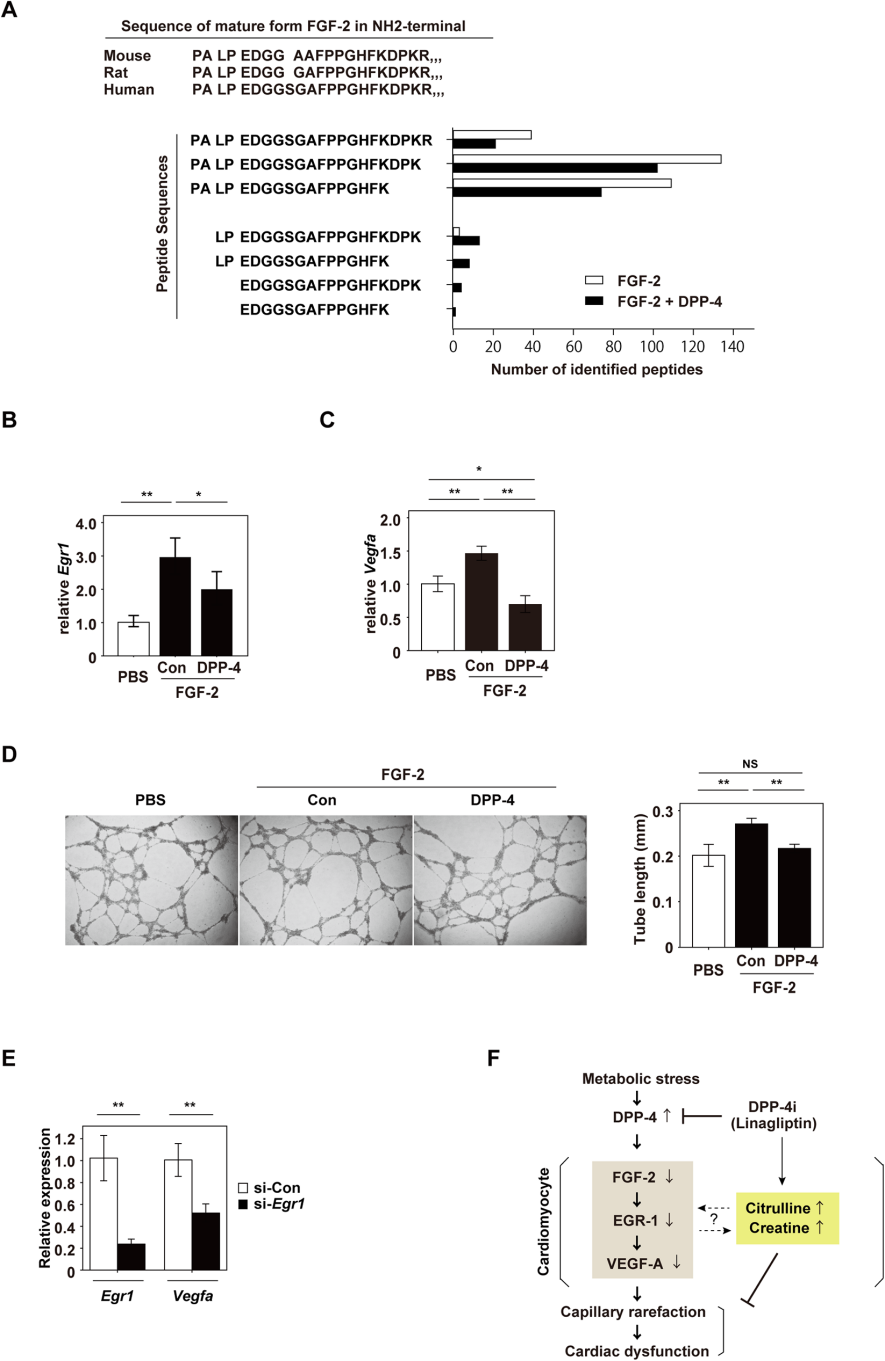
DPP-4 inhibits angiogenesis by suppressing FGF-2/EGR-1/VEGF-A signaling. (A) Known peptide sequences of the NH2-terminal of mature FGF-2 in mice, rats, and humans (upper panel). Lower panel indicates peptide sequences of the NH2-terminal of mature FGF-2 after incubation with DPP-4 (analyzed by LC-MS/MS). (B, C) Quantitative PCR for *Egr1* and *Vegfa* in neonatal rat ventricular myocytes (NRVMs) incubated with PBS, recombinant FGF-2 (FGF-2)(50 ng/ml, 2 hr), or DPP-4 (0.1 μg/ml, 2 hr) in Fig 4B (n = 4,4,4), and with FGF-2 (50 ng/ml, 8 hr) or DPP-4 (0.1 μg/ml, 8 hr) in Fig 4C(n = 4,4,4)). As for experiments in Fig 4B and C, FGF-2 was pre-incubated with DPP-4 in tube for totally 8 hours at 37°C before administration to cells in the FGF-2+DPP-4 group. (D) Tube formation by human umbilical vein endothelial cells (HUVECs) incubated with PBS or recombinant FGF-2 (FGF-2) (35 ng/ml for 12 hr), either with DPP-4 (0.1 μg/ml for 12 hr) or without DPP-4 (Con). Right panel indicates quantification of tube length for experiments shown in the left panel (n = 3,3,3). (E) Quantitative PCR for *Egr1* and *Vegfa* expression in neonatal rat ventricular myocytes (NRVMs) after introduction of control si-RNA (si-Con) or si-Egr1 (n = 4,4). (F) Scheme showing a summary of the present findings. Metabolic stress increases circulating DPP-4, which suppresses FGF-2/EGR-1/VEGF-A signaling in cardiac tissue, leading to capillary rarefaction and cardiac dysfunction. In addition to blocking DPP-4, linagliptin, a DPP-4 inhibitor (DPP-4i), increased citrulline and creatine levels in cardiac tissue, which may also have contributed to suppressing pathologic changes in the obese failing heart. Whether there is a link between altered metabolic profile and angiogenic response remains to be determined. Data were analyzed by the 2-tailed Student’s t-test (E), or 2-way ANOVA followed by Tukey’s multiple comparison test (B, C, D). *P<0.05, **P<0.01. All values represent the mean ± s.e.m. NS = not significant.

Previous studies have indicated that fibroblast growth factor 2 (FGF-2) activates EGR-1 [34, 35], and EGR-1 promotes angiogenesis via induction of vascular endothelial growth factor (VEGF) [33]. These reports promoted us to test whether FGF-2 is involved in regulation of EGR-1 in our model of dietary obesity. The peptide sequence of mature FGF-2 is conserved among mice, rats, and humans, and the NH2-terminal dipeptides of mature FGF-2 contain a putative truncation site for DPP-4 beginning with PALPEDGG, (Fig 4A; upper panel). Therefore, we tested whether DPP-4 actually cleaves the NH2-terminal dipeptides of FGF-2. LC-MS/MS demonstrated that co-incubation of FGF-2 with DPP-4 led to a decrease of mature FGF-2 along with an increase of cleaved FGF-2 commencing with LPEDGG, (Fig 4A; lower panel). The cleaved form of FGF-2 (LPEDGG,) also has a truncation site, and we found an increase of another truncated form of FGF-2 commencing with EDGG, which probably had been processed twice by DPP-4 (Fig 4A; lower panel). Incubation of neonatal rat ventricular myocytes (NRVMs) with FGF-2 led to an increase of *Egr1* and *Vegfa*, which was suppressed by addition of recombinant DPP-4 protein (Fig 4B and 4C). FGF-2 promoted tube formation by human umbilical vein endothelial cells (HUVECs) and this effect was suppressed by DPP-4 treatment (Fig 4D). We also found that genetic depletion of *Egr1* led to reduced *Vegfa* expression in NRVMs (Fig 4E). Taken together, these results indicate that FGF-2/EGR-1/VEGF-A signaling is involved in capillary network formation in the heart, and that DPP-4-induced pathology is partly mediated by suppression of this pathway through cleavage of mature FGF-2, leading to inhibition of angiogenesis.

## Discussion

DPP-4 has multiple complex biological effects, and it influences various molecules and pathways. The present study demonstrated that activation of angiogenesis, as well as an increase of citrulline and/or creatine levels, may contribute to the maintenance of cardiac homeostasis despite metabolic stress caused by dietary obesity. Citrulline can increase the availability of nitrate and nitric oxide (NO) [36, 37], and thus citrulline is expected to improve the delivery of oxygen in heart failure [38]. Citrulline administration is reported to suppress pathologic changes of diabetic cardiomyopathy in a genetic obesity model[39]. Citrulline was also reported to improve right ventricular function in patients with heart failure and a preserved ejection fraction [39, 40], while creatine was shown to reduce left ventricular dysfunction in models of left ventricular pressure overload and myocardial infarction [41]. Myocardial creatine depletion was reported to result in left ventricular dysfunction [42], while moderate elevation of creatine protected mice against acute myocardial infarction [43]. Moreover, addition of creatine to hypoxic cultured cardiac cells partially preserved mitochondrial creatine kinase activity [44].

Our present results also suggested that DPP-4 inhibits FGF-2/EGR-1/VEGF-A signaling to promote cardiac capillary rarefaction and cardiac dysfunction in mice with dietary obesity. As far as we know, this is the first report that DPP-4 cleaves NH2-terminal dipeptides of mature FGF-2. FGF-2 is processed into its mature form by truncation of 9 amino acids at the NH2-terminal (MAASGITSLPALPEDGG,), and there is another putative truncation site for DPP-4 following PALPEDGG,. It was reported that FGF-2 has a cardioprotective role by promoting angiogenesis in cardiac tissue [45], suggesting that suppression of FGF-2-induced angiogenesis by DPP-4 may be one of the pathologic changes mediated by this molecule.

In the present study, pharmacological inhibition of DPP-4 by linagliptin led to an increase of cardiac citrulline and creatine levels in mice with dietary obesity. Whether there is an interaction between alterations of the metabolic profile and the angiogenic response remains to be explored, and further studies are needed to identify the mechanisms underlying the increase of citrulline and creatine. Despite these limitations, our results demonstrate the cardioprotective effect of DPP-4 inhibitor therapy in the setting of heart failure associated with systemic metabolic dysfunction (Fig 4F).

## Supporting information

S1 Fig Effect of linagliptin on systemic glucose metabolism. (A) Oral glucose tolerance test (OGTT) or insulin tolerance test (ITT) in mice fed normal chow (NC), a high fat diet (HFD), or an HFD+linagliptin, a DPP-4 inhibitor (HFD+DPP-4i) (n = 5,5,6 for OGTT and n = 8,8,12 for ITT). (B) Systolic blood pressure (sBP) and diastolic blood pressure (dBP) of the indicated mice (n = 3,4,4). (C) Food intake of mice as indicated (n = 3,3,3). Data were analyzed by 2-way ANOVA followed by Tukey’s multiple comparison (A, B, C). **P<0.01 (NC vs HFD), ^**##**^*P*<0.01 (NC vs HFD+DPP-4i), ^**$ $**^*P*<0.01 (HFD vs HFD+DPP-4i). All values represent the mean ± s.e.m. NS = not significant.
(DOCX)

S2 Fig Characterization of metabolites in cardiac tissue. (A-C, E) Results of metabolomic analyses showing ATP (A), tyrosine and norepinephrine (B), glutamate, methionine and tryptophan (C), and UDP-glucuronate (E) in the cardiac tissue of mice fed normal chow (NC), a high fat diet (HFD), or an HFD+linagliptin, a DPP-4 inhibitor (HFD+DPP-4i) (n = 5,5,5). (D) Pathway enrichment analysis of cardiac tissue using metabolomic data. KEGG categories enriched by 1.5-fold in HFD mice (n = 5) compared to HFD+DPP-4i mice (n = 5) are described. Data were analyzed by 2-tailed Student’s *t*-test (D), with 2-way ANOVA followed by Tukey’s multiple comparison (A–C, E). *P<0.05, **P<0.01. All values represent the mean ± s.e.m. NS = not significant.
(DOCX)

S3 Fig Characterization of transcripts reduced in the cardiac tissue of dietary obese mice treated with linagliptin. In DNA microarray analysis, transcripts with GO terms such as “Angiogenesis”, “Vessel” or “Hypoxia” were extracted. Molecules showing lower expression in HFD+DPP-4i mice compared to untreated HFD mice are displayed [normal chow (NC) (n = 3), HFD (n = 3) and HFD+DPP-4i (n = 3)]. Data were analyzed by the 2-tailed Student’s t-test.
(DOCX)

S4 Fig Potential role of DPP-4 in the inhibition of angiogenesis in fibroblasts. (A) Quantitative PCR for *Vegfa* in neonatal rat fibroblasts incubated with FGF-2 (50 ng/ml, 12 hr) or DPP-4 (0.1 μg/ml, 12 hr) (n = 4,4,4)). In this study, FGF-2 was pre-incubated with DPP-4 in tube for totally 8hours at 37°C before administration to cells in FGF-2+DPP-4 group. (B) Quantitative PCR for *Egr1* and *Vegfa* expression in neonatal rat fibroblasts after introduction of control si-RNA (si-Con) or si-Egr1 (n = 4,4). Data were analyzed by the 2-tailed Student’s t-test (B), or 2-way ANOVA followed by Tukey’s multiple comparison test (A). *P<0.05, **P<0.01. All values represent the mean ± s.e.m. NS = not significant.
(DOCX)

S1 Table Cationic metabolites. Other cationic metabolites in the cardiac tissues of mice fed on a normal chow (NC) (n = 3), high fat diet (HF) (n = 3) and high fat diet+linagliptin (HF+Lina)(n = 3) groups. Data were analyzed by the 2-tailed Student’s t-test.
(DOCX)

S2 Table Anionic metabolites. Other anionis metabolites in the cardiac tissues of mice fed on a normal chow (NC) (n = 3), high fat diet (HF) (n = 3) and high fat diet+linagliptin (HF+Lina)(n = 3) groups. Data were analyzed by the 2-tailed Student’s t-test.
(DOCX)

S1 File ARRIVE Guideline checklist. These are the checklists for ARRIVE Guideline.
(PDF)

S2 File DNA microarray data of cardiac tissues. DNA microarray data of cardiac tissues of mice fed on a normal chow (NC) (n = 3), high fat diet (HF) (n = 3) and high fat diet+linagliptin (Lina)(n = 3) groups. Data were analyzed by the 2-tailed Student’s t-test.
(XLSX)

## References

[1] Ussher JR, Drucker DJ. Cardiovascular actions of incretin-based therapies. Circ Res. 2014;114(11):1788–803. doi: 10.1161/CIRCRESAHA.114.301958 . 24855202 10.1161/CIRCRESAHA.114.301958

[2] Zhong J, Maiseyeu A, Davis SN, Rajagopalan S. DPP4 in cardiometabolic disease: recent insights from the laboratory and clinical trials of DPP4 inhibition. Circ Res. 2015;116(8):1491–504. doi: 10.1161/CIRCRESAHA.116.305665 ; PubMed Central PMCID: PMC4394189. 25858071 10.1161/CIRCRESAHA.116.305665 PMC4394189

[3] Ou X, O'Leary HA, Broxmeyer HE. Implications of DPP4 modification of proteins that regulate stem/progenitor and more mature cell types. Blood. 2013;122(2):161–9. doi: 10.1182/blood-2013-02-487470 23637126 10.1182/blood-2013-02-487470 PMC3709652

[4] Mulvihill EE, Drucker DJ. Pharmacology, physiology, and mechanisms of action of dipeptidyl peptidase-4 inhibitors. Endocrine reviews. 2014;35(6):992–1019. doi: 10.1210/er.2014-1035 . 25216328 10.1210/er.2014-1035 PMC7108477

[5] Drucker DJ, Nauck MA. The incretin system: glucagon-like peptide-1 receptor agonists and dipeptidyl peptidase-4 inhibitors in type 2 diabetes. Lancet. 2006;368(9548):1696–705. doi: 10.1016/S0140-6736(06)69705-5 . 17098089 10.1016/S0140-6736(06)69705-5

[6] Sell H, Bluher M, Kloting N, Schlich R, Willems M, Ruppe F, et al Adipose Dipeptidyl Peptidase-4 and Obesity Correlation with insulin resistance and depot-specific release from adipose tissue in vivo and in vitro. Diabetes Care. 2013;36(12):4083–90. doi: 10.2337/dc13-0496 24130353 10.2337/dc13-0496 PMC3836153

[7] dos Santos L, Salles TA, Arruda-Junior DF, Campos LC, Pereira AC, Barreto AL, et al Circulating dipeptidyl peptidase IV activity correlates with cardiac dysfunction in human and experimental heart failure. Circ Heart Fail. 2013;6(5):1029–38. doi: 10.1161/CIRCHEARTFAILURE.112.000057 . 23894014 10.1161/CIRCHEARTFAILURE.112.000057

[8] Gomez N, Matheeussen V, Damoiseaux C, Tamborini A, Merveille AC, Jespers P, et al Effect of heart failure on dipeptidyl peptidase IV activity in plasma of dogs. J Vet Intern Med. 2012;26(4):929–34. doi: 10.1111/j.1939-1676.2012.00942.x . 22594653 10.1111/j.1939-1676.2012.00942.x

[9] Ingelsson E, Sundstrom J, Arnlov J, Zethelius B, Lind L. Insulin resistance and risk of congestive heart failure. JAMA. 2005;294(3):334–41. doi: 10.1001/jama.294.3.334 . 16030278 10.1001/jama.294.3.334

[10] Witteles RM, Fowler MB. Insulin-resistant cardiomyopathy clinical evidence, mechanisms, and treatment options. J Am Coll Cardiol. 2008;51(2):93–102. doi: 10.1016/j.jacc.2007.10.021 . 18191731 10.1016/j.jacc.2007.10.021

[11] Arnlov J, Lind L, Zethelius B, Andren B, Hales CN, Vessby B, et al Several factors associated with the insulin resistance syndrome are predictors of left ventricular systolic dysfunction in a male population after 20 years of follow-up. American Heart Journal. 2001;142(4):720–4. doi: 10.1067/mhj.2001.116957 11579365 10.1067/mhj.2001.116957

[12] Shimizu I, Minamino T, Toko H, Okada S, Ikeda H, Yasuda N, et al Excessive cardiac insulin signaling exacerbates systolic dysfunction induced by pressure overload in rodents. J Clin Invest. 2010;120(5):1506–14. Epub 2010/04/22. 40096 [pii] doi: 10.1172/JCI40096 ; PubMed Central PMCID: PMC2860916. 20407209 10.1172/JCI40096 PMC2860916

[13] Shimizu I, Yoshida Y, Katsuno T, Tateno K, Okada S, Moriya J, et al p53-induced adipose tissue inflammation is critically involved in the development of insulin resistance in heart failure. Cell Metab. 2012;15(1):51–64. Epub 2012/01/10. S1550-4131(11)00465-7 [pii] doi: 10.1016/j.cmet.2011.12.006 . 22225876 10.1016/j.cmet.2011.12.006

[14] Neubauer S. Mechanisms of disease—The failing heart—An engine out of fuel. New England Journal of Medicine. 2007;356(11):1140–51. doi: 10.1056/NEJMra063052 17360992 10.1056/NEJMra063052

[15] Huss JM, Kelly DP. Mitochondrial energy metabolism in heart failure: a question of balance. Journal of Clinical Investigation. 2005;115(3):547–55. doi: 10.1172/JCI200524405 15765136 10.1172/JCI200524405 PMC1052011

[16] Doenst T, Nguyen TD, Abel ED. Cardiac metabolism in heart failure: implications beyond ATP production. Circ Res. 2013;113(6):709–24. doi: 10.1161/CIRCRESAHA.113.300376 ; PubMed Central PMCID: PMC3896379. 23989714 10.1161/CIRCRESAHA.113.300376 PMC3896379

[17] Shigeta T, Aoyama M, Bando YK, Monji A, Mitsui T, Takatsu M, et al Dipeptidyl peptidase-4 modulates left ventricular dysfunction in chronic heart failure via angiogenesis-dependent and -independent actions. Circulation. 2012;126(15):1838–51. doi: 10.1161/CIRCULATIONAHA.112.096479 . 23035207 10.1161/CIRCULATIONAHA.112.096479

[18] Gomez N, Touihri K, Matheeussen V, Mendes Da Costa A, Mahmoudabady M, Mathieu M, et al Dipeptidyl peptidase IV inhibition improves cardiorenal function in overpacing-induced heart failure. Eur J Heart Fail. 2012;14(1):14–21. doi: 10.1093/eurjhf/hfr146 . 22045924 10.1093/eurjhf/hfr146

[19] Takahashi A, Asakura M, Ito S, Min KD, Shindo K, Yan Y, et al Dipeptidyl-peptidase IV inhibition improves pathophysiology of heart failure and increases survival rate in pressure-overloaded mice. Am J Physiol Heart Circ Physiol. 2013;304(10):H1361–9. doi: 10.1152/ajpheart.00454.2012 . 23504176 10.1152/ajpheart.00454.2012

[20] Bostick B, Habibi J, Ma L, Aroor A, Rehmer N, Hayden MR, et al Dipeptidyl peptidase inhibition prevents diastolic dysfunction and reduces myocardial fibrosis in a mouse model of Western diet induced obesity. Metabolism. 2014;63(8):1000–11. doi: 10.1016/j.metabol.2014.04.002 ; PubMed Central PMCID: PMC4128682. 24933400 10.1016/j.metabol.2014.04.002 PMC4128682

[21] Gilbert RE, Krum H. Heart failure in diabetes: effects of anti-hyperglycaemic drug therapy. Lancet. 2015;385(9982):2107–17. doi: 10.1016/S0140-6736(14)61402-1 26009231 10.1016/S0140-6736(14)61402-1

[22] Scirica BM, Bhatt DL, Braunwald E, Steg PG, Davidson J, Hirshberg B, et al Saxagliptin and cardiovascular outcomes in patients with type 2 diabetes mellitus. N Engl J Med. 2013;369(14):1317–26. doi: 10.1056/NEJMoa1307684 . 23992601 10.1056/NEJMoa1307684

[23] Zannad F, Cannon CP, Cushman WC, Bakris GL, Menon V, Perez AT, et al Heart failure and mortality outcomes in patients with type 2 diabetes taking alogliptin versus placebo in EXAMINE: a multicentre, randomised, double-blind trial. Lancet. 2015;385(9982):2067–76. doi: 10.1016/S0140-6736(14)62225-X . 25765696 10.1016/S0140-6736(14)62225-X

[24] Green JB, Bethel MA, Armstrong PW, Buse JB, Engel SS, Garg J, et al Effect of Sitagliptin on Cardiovascular Outcomes in Type 2 Diabetes. N Engl J Med. 2015;373(3):232–42. doi: 10.1056/NEJMoa1501352 . 26052984 10.1056/NEJMoa1501352

[25] White WB, Cannon CP, Heller SR, Nissen SE, Bergenstal RM, Bakris GL, et al Alogliptin after acute coronary syndrome in patients with type 2 diabetes. N Engl J Med. 2013;369(14):1327–35. doi: 10.1056/NEJMoa1305889 . 23992602 10.1056/NEJMoa1305889

[26] Filion KB, Azoulay L, Platt RW, Dahl M, Dormuth CR, Clemens KK, et al A Multicenter Observational Study of Incretin-based Drugs and Heart Failure. N Engl J Med. 2016;374(12):1145–54. doi: 10.1056/NEJMoa1506115 . 27007958 10.1056/NEJMoa1506115

[27] Sano M, Minamino T, Toko H, Miyauchi H, Orimo M, Qin Y, et al p53-induced inhibition of Hif-1 causes cardiac dysfunction during pressure overload. Nature. 2007;446(7134):444–8. Epub 2007/03/06. doi: 10.1038/nature05602 . 17334357 10.1038/nature05602

[28] Hirayama A, Kami K, Sugimoto M, Sugawara M, Toki N, Onozuka H, et al Quantitative metabolome profiling of colon and stomach cancer microenvironment by capillary electrophoresis time-of-flight mass spectrometry. Cancer Res. 2009;69(11):4918–25. doi: 10.1158/0008-5472.CAN-08-4806 . 19458066 10.1158/0008-5472.CAN-08-4806

[29] Lu X, Zhu H. Tube-gel digestion: a novel proteomic approach for high throughput analysis of membrane proteins. Molecular & cellular proteomics: MCP. 2005;4(12):1948–58. doi: 10.1074/mcp.M500138-MCP200 ; PubMed Central PMCID: PMC1360194. 16150870 10.1074/mcp.M500138-MCP200 PMC1360194

[30] Katayama H, Nagasu T, Oda Y. Improvement of in-gel digestion protocol for peptide mass fingerprinting by matrix-assisted laser desorption/ionization time-of-flight mass spectrometry. Rapid communications in mass spectrometry: RCM. 2001;15(16):1416–21. doi: 10.1002/rcm.379 . 11507753 10.1002/rcm.379

[31] Storey JD, Tibshirani R. Statistical significance for genomewide studies. Proc Natl Acad Sci U S A. 2003;100(16):9440–5. doi: 10.1073/pnas.1530509100 ; PubMed Central PMCID: PMCPMC170937. 12883005 10.1073/pnas.1530509100 PMC170937

[32] Ortega FB, Lavie CJ, Blair SN. Obesity and Cardiovascular Disease. Circulation research. 2016;118(11):1752–70. doi: 10.1161/CIRCRESAHA.115.306883 . 27230640 10.1161/CIRCRESAHA.115.306883

[33] Shimoyamada H, Yazawa T, Sato H, Okudela K, Ishii J, Sakaeda M, et al Early growth response-1 induces and enhances vascular endothelial growth factor-A expression in lung cancer cells. Am J Pathol. 2010;177(1):70–83. doi: 10.2353/ajpath.2010.091164 ; PubMed Central PMCID: PMC2893652. 20489156 10.2353/ajpath.2010.091164 PMC2893652

[34] Silverman ES, Collins T. Pathways of Egr-1-mediated gene transcription in vascular biology. Am J Pathol. 1999;154(3):665–70. doi: 10.1016/S0002-9440(10)65312-6 ; PubMed Central PMCID: PMC1866415. 10079243 10.1016/S0002-9440(10)65312-6 PMC1866415

[35] Passiatore G, Gentilella A, Rom S, Pacifici M, Bergonzini V, Peruzzi F. Induction of Id-1 by FGF-2 involves activity of EGR-1 and sensitizes neuroblastoma cells to cell death. J Cell Physiol. 2011;226(7):1763–70. doi: 10.1002/jcp.22505 ; PubMed Central PMCID: PMC3760689. 21506108 10.1002/jcp.22505 PMC3760689

[36] Schwedhelm E, Maas R, Freese R, Jung D, Lukacs Z, Jambrecina A, et al Pharmacokinetic and pharmacodynamic properties of oral L-citrulline and L-arginine: impact on nitric oxide metabolism. British Journal of Clinical Pharmacology. 2008;65(1):51–9. doi: 10.1111/j.1365-2125.2007.02990.x 17662090 10.1111/j.1365-2125.2007.02990.x PMC2291275

[37] Morita M, Hayashi T, Ochiai M, Maeda M, Yamaguchi T, Ina K, et al Oral supplementation with a combination of L-citrulline and L-arginine rapidly increases plasma L-arginine concentration and enhances NO bioavailability. Biochemical and Biophysical Research Communications. 2014;454(1):53–7. doi: 10.1016/j.bbrc.2014.10.029 25445598 10.1016/j.bbrc.2014.10.029

[38] Alsop P, Hauton D. Oral nitrate and citrulline decrease blood pressure and increase vascular conductance in young adults: a potential therapy for heart failure. European Journal of Applied Physiology. 2016;116(9):1651–61. doi: 10.1007/s00421-016-3418-7 27333912 10.1007/s00421-016-3418-7 PMC4983290

[39] Baumgardt SL, Paterson M, Leucker TM, Fang J, Zhang DX, Bosnjak ZJ, et al Chronic Co-Administration of Sepiapterin and L-Citrulline Ameliorates Diabetic Cardiomyopathy and Myocardial Ischemia/Reperfusion Injury in Obese Type 2 Diabetic Mice. Circulation-Heart Failure. 2016;9(1). doi: 10.1161/Circheartfailure.115.002424 26763290 10.1161/CIRCHEARTFAILURE.115.002424 PMC4714787

[40] Orozco-Gutierrez JJ, Castillo-Martinez L, Orea-Tejeda A, Vazquez-Diaz O, Valdespino-Trejo A, Narvaez-David R, et al Effect of L-arginine or L-citrulline oral supplementation on blood pressure and right ventricular function in heart failure patients with preserved ejection fraction. Cardiol J. 2010;17(6):612–8. . 21154265

[41] Lygate CA, Fischer A, Sebag-Montefiore L, Wallis J, ten Hove M, Neubauer S. The creatine kinase energy transport system in the failing mouse heart. Journal of Molecular and Cellular Cardiology. 2007;42(6):1129–36. doi: 10.1016/j.yjmcc.2007.03.899 17481652 10.1016/j.yjmcc.2007.03.899

[42] Lindbom M, Ramunddal T, Camejo G, Waagstein F, Omerovic E. In vivo effects of myocardial creatine depletion on left ventricular function morphology and lipid metabolism: Study in a mouse model. Journal of Cardiac Failure. 2008;14(2):161–6. doi: 10.1016/j.cardfail.2007.10.020 18325464 10.1016/j.cardfail.2007.10.020

[43] Lygate CA, Bohl S, ten Hove M, Faller KME, Ostrowski PJ, Zervou S, et al Moderate elevation of intracellular creatine by targeting the creatine transporter protects mice from acute myocardial infarction. Cardiovascular Research. 2012;96(3):466–75. doi: 10.1093/cvr/cvs272 22915766 10.1093/cvr/cvs272 PMC3500046

[44] Millanvoyevanbrussel E, Freyssbeguin M, Griffaton G, Lechat P. Energy-Metabolism of Cardiac Cell-Cultures during Oxygen Deprivation—Effects of Creatine and Arachidonic-Acid. Biochemical Pharmacology. 1985;34(1):145–7. doi: 10.1016/0006-2952(85)90114-5 3917667 10.1016/0006-2952(85)90114-5

[45] Yajima S, Ishikawa M, Kubota T, Moroi M, Sugi K, Namiki A. Intramyocardial injection of fibroblast growth factor-2 plus heparin suppresses cardiac failure progression in rats with hypertensive heart disease. International Heart Journal. 2005;46(2):289–301. doi: 10.1536/Ihj.46.289 15876812 10.1536/ihj.46.289

