# Supplementary material for: Inhibition of dipeptidyl peptidase-4 ameliorates cardiac ischemia and systolic dysfunction by up-regulating the FGF-2/EGR-1 pathway
Source: PLoS One. 2017 Aug 3;12(8):e0182422. doi: 10.1371/journal.pone.0182422 (PMC5542565; doi:10.1371/journal.pone.0182422)
Supplement: S1 Fig — (A) Oral glucose tolerance test (OGTT) or insulin tolerance test (ITT) in mice fed normal chow (NC), a high fat diet (HFD), or an HFD+linagliptin, a DPP-4 inhibitor (HFD+DPP-4i) (n = 5,5,6 for OGTT and n = 8,8,12 for ITT). (B) Systolic blood pressure (sBP) and diastolic blood pressure (dBP) of the indicated mice (n = 3,4,4). (C) Food intake of mice as indicated (n = 3,3,3). Data were analyzed by 2-way ANOVA followed by Tukey’s multiple comparison (A, B, C). **P<0.01 (NC vs HFD), ##P<0.01 (NC vs HFD+DPP-4i), $ $P<0.01 (HFD vs HFD+DPP-4i). All values represent the mean ± s.e.m. NS = not significant. (DOCX) [file pone.0182422.s001.docx]

**S1 Fig Effect of linagliptin on systemic glucose metabolism**

(A) Oral glucose tolerance test (OGTT) or insulin tolerance test (ITT) in mice fed normal chow (NC), a high fat diet (HFD), or an HFD+linagliptin, a DPP-4 inhibitor (HFD+DPP-4i) (n=5,5,6 for OGTT and n=8,8,12 for ITT). (B) Systolic blood pressure (sBP) and diastolic blood pressure (dBP) of the indicated mice (n=3,4,4). (C) Food intake of mice as indicated (n=3,3,3). Data were analyzed by 2-way ANOVA followed by Tukey’s multiple comparison (A, B, C). ***P*<0.01 (NC vs HFD), **^##^***P*<0.01 (NC vs HFD+DPP-4i), **^$$^***P*<0.01 (HFD vs HFD+DPP-4i). All values represent the mean ± s.e.m. NS = not significant.
