# Supplementary material for: Inhibition of dipeptidyl peptidase-4 ameliorates cardiac ischemia and systolic dysfunction by up-regulating the FGF-2/EGR-1 pathway
Source: PLoS One. 2017 Aug 3;12(8):e0182422. doi: 10.1371/journal.pone.0182422 (PMC5542565; doi:10.1371/journal.pone.0182422)
Supplement: S2 Fig — (A-C, E) Results of metabolomic analyses showing ATP (A), tyrosine and norepinephrine (B), glutamate, methionine and tryptophan (C), and UDP-glucuronate (E) in the cardiac tissue of mice fed normal chow (NC), a high fat diet (HFD), or an HFD+linagliptin, a DPP-4 inhibitor (HFD+DPP-4i) (n = 5,5,5). (D) Pathway enrichment analysis of cardiac tissue using metabolomic data. KEGG categories enriched by 1.5-fold in HFD mice (n = 5) compared to HFD+DPP-4i mice (n = 5) are described. Data were analyzed by 2-tailed Student’s t-test (D), with 2-way ANOVA followed by Tukey’s multiple comparison (A–C, E). *P<0.05, **P<0.01. All values represent the mean ± s.e.m. NS = not significant. (DOCX) [file pone.0182422.s002.docx]

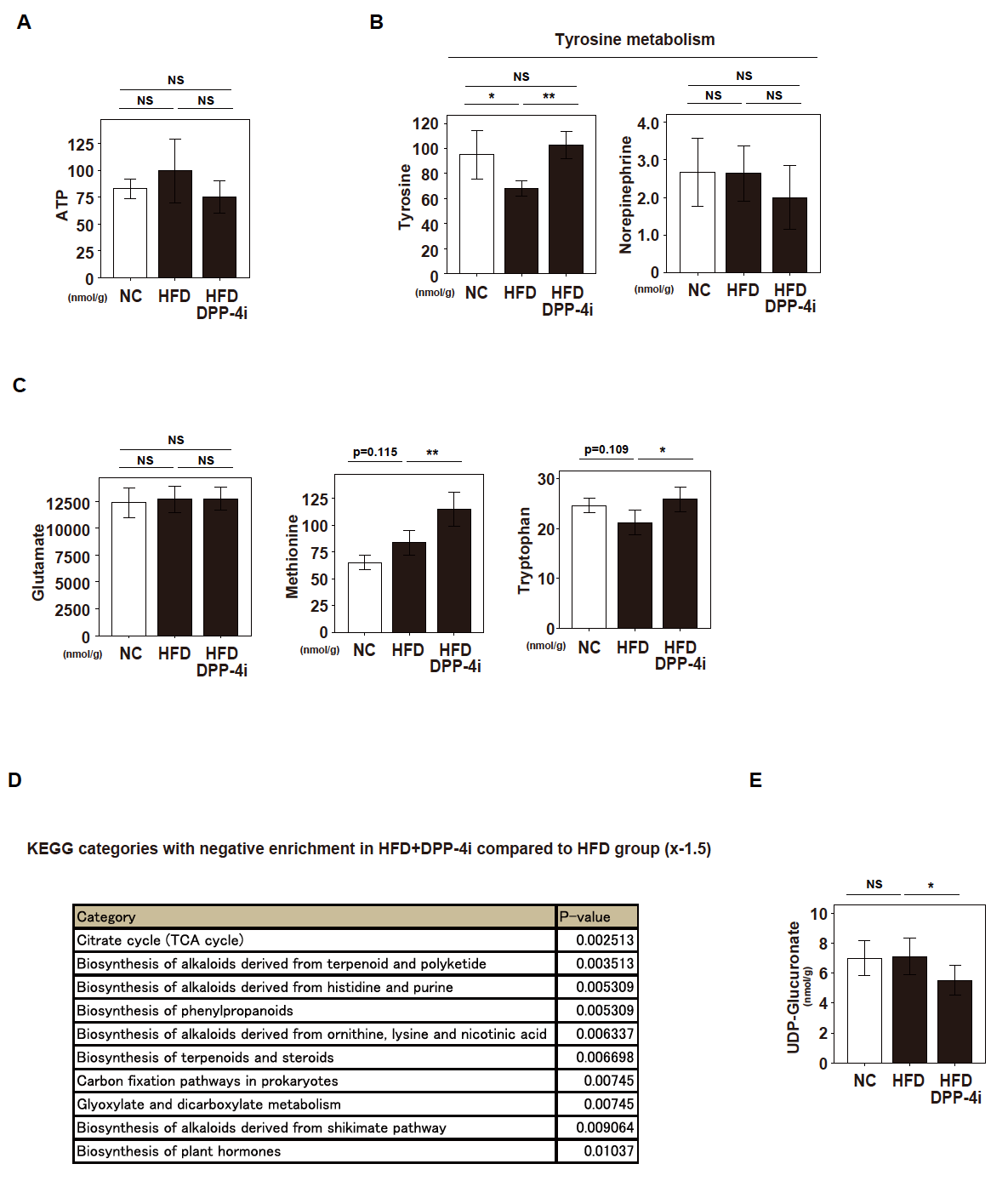
**S2 Fig Characterization of metabolites in cardiac tissue**

(A-C, E) Results of metabolomic analyses showing ATP (A), tyrosine and norepinephrine (B), glutamate, methionine and tryptophan (C), and UDP-glucuronate (E) in the cardiac tissue of mice fed normal chow (NC), a high fat diet (HFD), or an HFD+linagliptin, a DPP-4 inhibitor (HFD+DPP-4i) (n=5,5,5). (D) Pathway enrichment analysis of cardiac tissue using metabolomic data. KEGG categories enriched by 1.5-fold in HFD mice (n=5) compared to HFD+DPP-4i mice (n=5) are described. Data were analyzed by 2-tailed Student’s *t*-test (D), with 2-way ANOVA followed by Tukey’s multiple comparison (A–C, E). **P*<0.05, ***P*<0.01. All values represent the mean ± s.e.m. NS = not significant.
