# Supplementary material for: Inhibition of dipeptidyl peptidase-4 ameliorates cardiac ischemia and systolic dysfunction by up-regulating the FGF-2/EGR-1 pathway
Source: PLoS One. 2017 Aug 3;12(8):e0182422. doi: 10.1371/journal.pone.0182422 (PMC5542565; doi:10.1371/journal.pone.0182422)
Supplement: S3 Fig — In DNA microarray analysis, transcripts with GO terms such as “Angiogenesis”, “Vessel” or “Hypoxia” were extracted. Molecules showing lower expression in HFD+DPP-4i mice compared to untreated HFD mice are displayed [normal chow (NC) (n = 3), HFD (n = 3) and HFD+DPP-4i (n = 3)]. Data were analyzed by the 2-tailed Student’s t-test. (DOCX) [file pone.0182422.s003.docx]

**S3 Fig Characterization of transcripts reduced in the cardiac tissue of dietary obese mice treated with linagliptin**

**
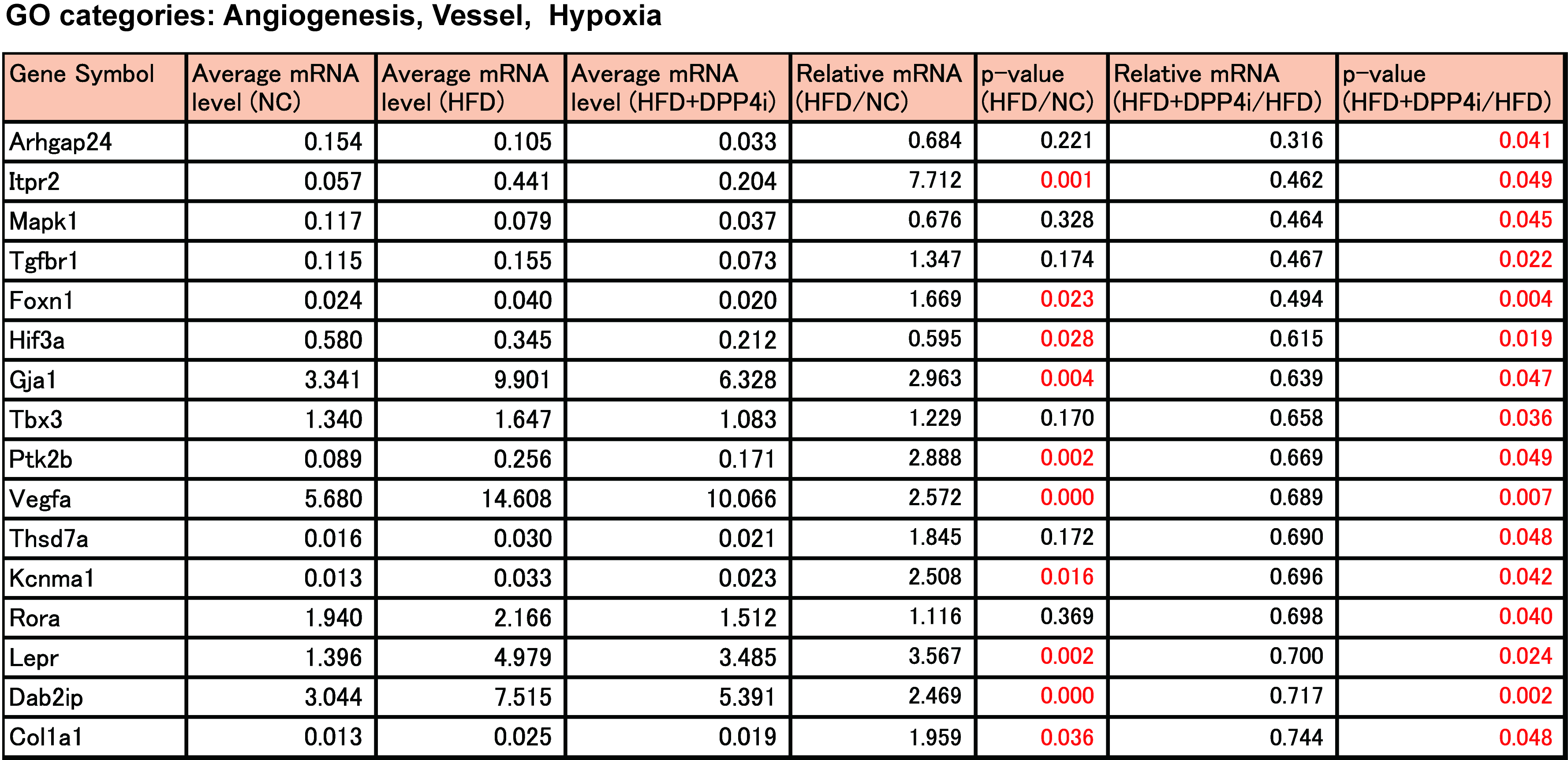
**

In DNA microarray analysis, transcripts with GO terms such as “Angiogenesis”, “Vessel” or “Hypoxia” were extracted. Molecules showing lower expression in HFD+DPP-4i mice compared to untreated HFD mice are displayed [normal chow (NC) (n=3), HFD (n=3) and HFD+DPP-4i (n=3)]. Data were analyzed by the 2-tailed Student’s t-test.
