# Supplementary material for: Inhibition of dipeptidyl peptidase-4 ameliorates cardiac ischemia and systolic dysfunction by up-regulating the FGF-2/EGR-1 pathway
Source: PLoS One. 2017 Aug 3;12(8):e0182422. doi: 10.1371/journal.pone.0182422 (PMC5542565; doi:10.1371/journal.pone.0182422)
Supplement: S4 Fig — (A) Quantitative PCR for Vegfa in neonatal rat fibroblasts incubated with FGF-2 (50 ng/ml, 12 hr) or DPP-4 (0.1 μg/ml, 12 hr) (n = 4,4,4)). In this study, FGF-2 was pre-incubated with DPP-4 in tube for totally 8hours at 37°C before administration to cells in FGF-2+DPP-4 group. (B) Quantitative PCR for Egr1 and Vegfa expression in neonatal rat fibroblasts after introduction of control si-RNA (si-Con) or si-Egr1 (n = 4,4). Data were analyzed by the 2-tailed Student’s t-test (B), or 2-way ANOVA followed by Tukey’s multiple comparison test (A). *P<0.05, **P<0.01. All values represent the mean ± s.e.m. NS = not significant. (DOCX) [file pone.0182422.s004.docx]

**S4 Fig Potential role of DPP-4 in the inhibition of angiogenesis in fibroblasts**

**
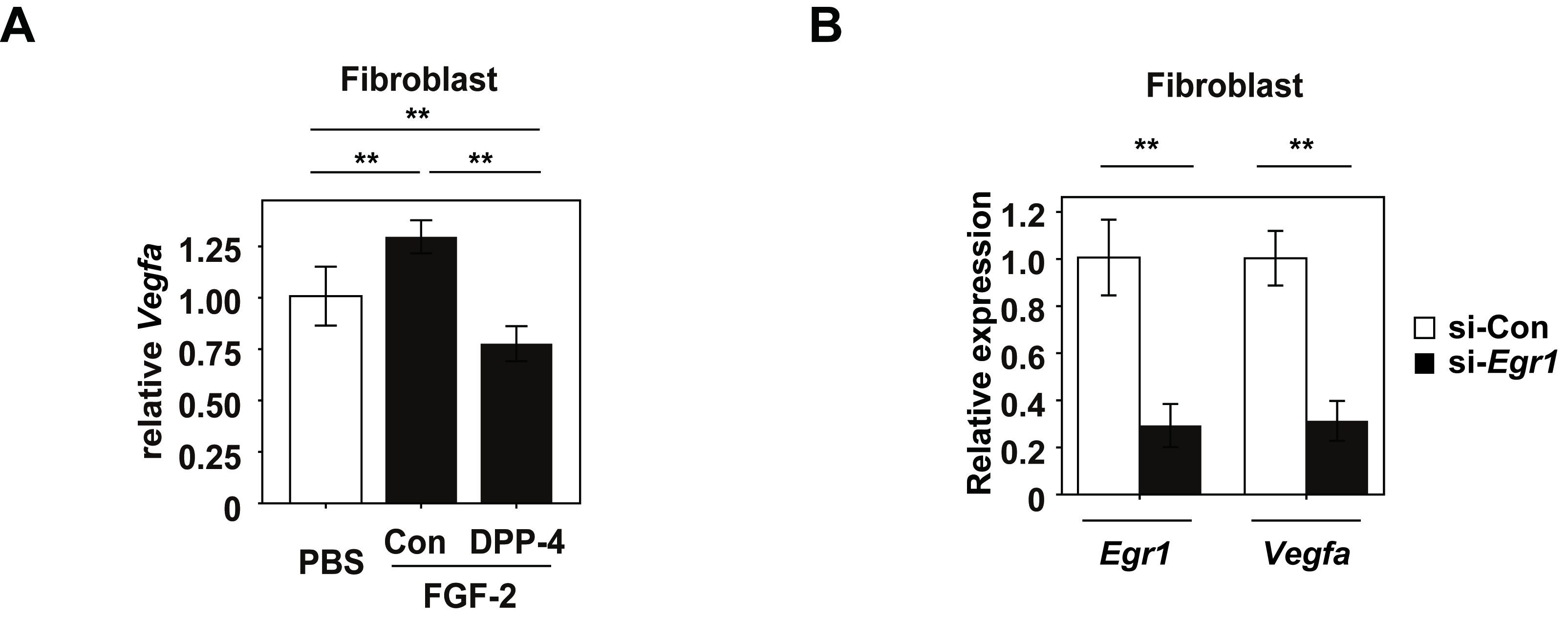
**

(A) Quantitative PCR for *Vegfa* in neonatal rat fibroblasts incubated with FGF-2 (50 ng/ml, 12 hr) or DPP-4 (0.1 μg/ml, 12 hr) (n=4,4,4)). In this study, FGF-2 was pre-incubated with DPP-4 in tube for totally 8hours at 37˚C before administration to cells in FGF-2+DPP-4 group. (B) Quantitative PCR for *Egr1* and *Vegfa* expression in neonatal rat fibroblasts after introduction of control si-RNA (si-Con) or si-Egr1 (n=4,4). Data were analyzed by the 2-tailed Student’s t-test (B), or 2-way ANOVA followed by Tukey’s multiple comparison test (A). *P<0.05, **P<0.01. All values represent the mean ± s.e.m. NS = not significant.
