# Supplementary material for: Inhibition of dipeptidyl peptidase-4 ameliorates cardiac ischemia and systolic dysfunction by up-regulating the FGF-2/EGR-1 pathway
Source: PLoS One. 2017 Aug 3;12(8):e0182422. doi: 10.1371/journal.pone.0182422 (PMC5542565; doi:10.1371/journal.pone.0182422)
Supplement: S1 Table — Other cationic metabolites in the cardiac tissues of mice fed on a normal chow (NC) (n = 3), high fat diet (HF) (n = 3) and high fat diet+linagliptin (HF+Lina)(n = 3) groups. Data were analyzed by the 2-tailed Student’s t-test. (DOCX) [file pone.0182422.s005.docx]

**Table S1 Cationic metabolites**

| nmol/g (Cardiac tissue weight) | |  |  |  |  |  |  |  |  |  |  |  |  |  |  |  |  |  |  |
| --- | --- | --- | --- | --- | --- | --- | --- | --- | --- | --- | --- | --- | --- | --- | --- | --- | --- | --- | --- |
|  |  | Group | | **NC** | | | | | **HF** | | | | | **HF+Lina** | | | | | **HF vs HF+LIna (StudentT)** |
|  |  | Tube no. | | NC1 | NC2 | NC3 | NC4 | NC5 | HF1 | HF2 | HF3 | HF4 | HF5 | HF+Lina1 | HF+Lina2 | HF+Lina3 | HF+Lina4 | HF+Lina5 |  |
|  |  | Ave m/z | Ave CorMT |  |  |  |  |  |  |  |  |  |  |  |  |  |  |  |  |
| [C05127](http://www.genome.jp/dbget-bin/www_bget?cpd:C05127) | 1-Methylhistamine | 126.1022 | 7.05 | 2.1 | 2.4 | 3.4 | 3.6 | 4.3 | 3.5 | 2.0 | 1.9 | 2.7 | 3.2 | 3.4 | 3.2 | 3.6 | 2.5 | 4.9 | p=NS |
| [C02918](http://www.genome.jp/dbget-bin/www_bget?cpd:C02918) | 1-Methylnicotinamide | 137.0707 | 10.57 | 1.1 | 1.1 | 1.5 | 1.4 | 1.4 | 1.6 | 2.6 | 2.0 | 1.8 | 4.1 | 1.8 | 1.4 | 3.2 | 1.2 | 2.4 | p=NS |
| [C00881](http://www.genome.jp/dbget-bin/www_bget?cpd:C00881) | 2'-Deoxycytidine | 228.1000 | 13.61 | 1.9 | N.D. | N.D. | N.D. | 1.8 | 2.2 | 1.4 | 1.5 | N.D. | N.D. | 2.0 | N.D. | N.D. | 1.9 | 2.0 | p=NS |
| [C02356](http://www.genome.jp/dbget-bin/www_bget?cpd:C02356) | 2AB | 104.0694 | 13.93 | 10 | 8.6 | 7.3 | 6.0 | 6.8 | 5.5 | 7.9 | 6.4 | 6.6 | 8.0 | 6.7 | 9.3 | 5.3 | 5.2 | 6.5 | p=NS |
| [C01152](http://www.genome.jp/dbget-bin/www_bget?cpd:C01152) | 3-Methylhistidine | 170.0930 | 10.78 | 12 | 13 | 15 | 14 | 15 | 7.3 | 8.6 | 9.3 | 9.1 | 8.9 | 6.4 | 8.2 | 11 | 11 | 8.5 | p=NS |
| [C16741](http://www.genome.jp/dbget-bin/www_bget?cpd:C16741) | 5-Hydroxylysine | 163.1074 | 10.29 | 1.4 | 1.4 | 1.9 | 1.5 | 1.5 | 0.79 | 0.73 | 0.60 | 0.96 | N.D. | 0.77 | 0.73 | 0.85 | N.D. | N.D. | p=NS |
| [C00170](http://www.genome.jp/dbget-bin/www_bget?cpd:C00170) | 5-Methylthioadenosine | 298.0982 | 14.58 | 3.6 | 4.8 | 4.1 | 3.7 | 4.3 | 4.4 | 4.6 | 4.3 | 4.7 | 3.9 | 3.8 | 4.4 | 4.1 | 4.3 | 3.6 | p=NS |
| [C01996](http://www.genome.jp/dbget-bin/www_bget?cpd:C01996) | Acetylcholine | 146.1176 | 10.96 | 1.0 | 1.7 | 1.5 | 1.0 | 0.62 | 0.88 | 1.0 | 1.3 | 0.51 | 0.93 | 0.57 | 0.63 | 1.2 | 0.63 | 0.74 | p=NS |
| [C00147](http://www.genome.jp/dbget-bin/www_bget?cpd:C00147) | Adenine | 136.0603 | 10.97 | 8.3 | 6.9 | 6.7 | 8.1 | 7.9 | 8.2 | 5.7 | 8.1 | 7.5 | 9.3 | 8.0 | 8.1 | 7.5 | 7.2 | 8.0 | p=NS |
| [C00212](http://www.genome.jp/dbget-bin/www_bget?cpd:C00212) | Adenosine | 268.1061 | 14.24 | 207 | 130 | 196 | 298 | 228 | 276 | 131 | 252 | 285 | 307 | 196 | 253 | 202 | 246 | 218 | p=NS |
| [C03626](http://www.genome.jp/dbget-bin/www_bget?cpd:C03626) | ADMA | 203.1497 | 11.05 | 4.0 | 3.9 | 3.4 | 3.3 | 3.4 | 2.3 | 3.1 | 3.1 | 3.4 | 3.0 | 2.7 | 2.3 | 3.2 | 4.0 | 2.6 | p=NS |
| [C04677](http://www.genome.jp/dbget-bin/www_bget?cpd:C04677) | AICAR | 339.0722 | 32.75 | 5.9 | N.D. | N.D. | N.D. | N.D. | N.D. | 4.3 | N.D. | N.D. | N.D. | 7.2 | N.D. | 7.2 | 5.5 | N.D. | p=NS |
| [C00041](http://www.genome.jp/dbget-bin/www_bget?cpd:C00041) | Ala | 90.0551 | 13.01 | 1311 | 1615 | 1944 | 1479 | 1879 | 2824 | 2633 | 2799 | 2986 | 3007 | 2989 | 3220 | 3368 | 2294 | 3334 | p=NS |
| [C01551](http://www.genome.jp/dbget-bin/www_bget?cpd:C01551) | Allantoin | 159.0532 | 32.24 | 131 | 132 | 164 | 152 | 178 | 113 | 119 | 93 | 180 | 135 | 140 | 114 | 149 | 129 | 115 | p=NS |
| [C00956](http://www.genome.jp/dbget-bin/www_bget?cpd:C00956) | alpha-Aminoadipate | 162.0757 | 15.84 | 6.9 | 6.7 | 6.7 | 6.9 | 7.0 | 4.5 | 4.8 | 4.8 | 6.3 | 5.1 | 5.6 | 5.5 | 4.4 | 4.7 | 5.3 | p=NS |
| [C02115](http://www.genome.jp/dbget-bin/www_bget?cpd:C02115) | Alpha-Methylserine | 120.0640 | 14.98 | 2.1 | 1.4 | 2.2 | 2.4 | 3.0 | N.D. | N.D. | N.D. | N.D. | N.D. | N.D. | N.D. | N.D. | N.D. | N.D. | p=NS |
| [C00062](http://www.genome.jp/dbget-bin/www_bget?cpd:C00062) | Arg | 175.1203 | 10.23 | 343 | 321 | 293 | 300 | 333 | 293 | 348 | 394 | 359 | 281 | 343 | 244 | 375 | 386 | 368 | p=NS |
| [C03406](http://www.genome.jp/dbget-bin/www_bget?cpd:C03406) | Argininosuccinate | 291.1318 | 13.60 | 7.3 | 4.4 | 4.9 | 4.4 | 6.5 | 6.0 | 3.8 | 3.3 | 5.2 | 4.2 | 6.2 | 4.5 | 5.4 | 5.7 | 7.7 | p=NS |
| [C00152](http://www.genome.jp/dbget-bin/www_bget?cpd:C00152) | Asn | 133.0607 | 15.11 | 321 | 355 | 275 | 235 | 279 | 336 | 424 | 396 | 389 | 345 | 406 | 324 | 412 | 423 | 427 | p=NS |
| [C00049](http://www.genome.jp/dbget-bin/www_bget?cpd:C00049) | Asp | 134.0448 | 16.68 | 1802 | 1568 | 2676 | 1828 | 2211 | 2185 | 3964 | 3510 | 3040 | 2430 | 2980 | 1839 | 3038 | 1923 | 1932 | p=NS |
| [C00099](http://www.genome.jp/dbget-bin/www_bget?cpd:C00099) | beta-Ala | 90.0541 | 10.54 | 19 | 27 | 19 | 23 | 25 | 32 | 32 | 31 | 32 | 28 | 32 | 26 | 28 | 30 | 31 | p=NS |
| [C00719](http://www.genome.jp/dbget-bin/www_bget?cpd:C00719) | Betaine | 118.0852 | 16.31 | 150 | 162 | 107 | 99 | 115 | 54 | 53 | 76 | 62 | 65 | 57 | 56 | 63 | 91 | 64 | p=NS |
| [C00318](http://www.genome.jp/dbget-bin/www_bget?cpd:C00318) | Carnitine | 162.1146 | 12.24 | 1895 | 1947 | 1906 | 1826 | 2186 | 1219 | 1155 | 1274 | 1220 | 1216 | 1184 | 1117 | 1287 | 1166 | 1048 | p=NS |
| [C00386](http://www.genome.jp/dbget-bin/www_bget?cpd:C00386) | Carnosine | 114.0598 | 9.73 | 33 | 36 | 26 | 21 | 31 | 27 | 31 | 29 | 30 | 24 | 28 | 22 | 31 | 34 | 26 | p=NS |
| [C00114](http://www.genome.jp/dbget-bin/www_bget?cpd:C00114) | Choline | 104.1065 | 9.85 | 296 | 260 | 251 | 234 | 276 | 297 | 188 | 173 | 291 | 211 | 323 | 234 | 251 | 314 | 268 | p=NS |
| [C00791](http://www.genome.jp/dbget-bin/www_bget?cpd:C00791) | Creatinine | 114.0645 | 10.50 | 47 | 62 | 57 | 57 | 60 | 71 | 41 | 48 | 58 | 89 | 66 | 65 | 68 | 59 | 59 | p=NS |
| [C02291](http://www.genome.jp/dbget-bin/www_bget?cpd:C02291) | Cystathionine | 223.0756 | 14.30 | 1.1 | N.D. | 1.6 | N.D. | 1.8 | 1.4 | 1.4 | 1.6 | N.D. | 1.7 | 1.9 | 1.8 | 1.9 | N.D. | 1.8 | p=NS |
| [C00475](http://www.genome.jp/dbget-bin/www_bget?cpd:C00475) | Cytidine | 244.0956 | 13.97 | 60 | 40 | 43 | 48 | 52 | 62 | 30 | 39 | 58 | 59 | 72 | 57 | 55 | 61 | 73 | p=NS |
| [C06772](http://www.genome.jp/dbget-bin/www_bget?cpd:C06772) | Diethanolamine | 106.0848 | 11.01 | 43 | 9.4 | 6.4 | 10 | 27 | 35 | 13 | 8.1 | 42 | 7.0 | 4.7 | 8.8 | 5.6 | 6.7 | 16 | p=NS |
| [C00334](http://www.genome.jp/dbget-bin/www_bget?cpd:C00334) | GABA | 104.0694 | 11.07 | 8.9 | 6.3 | 5.4 | 10 | 5.9 | 3.7 | 2.6 | 1.8 | 3.5 | 4.2 | 2.8 | 4.3 | 3.4 | 3.7 | 3.4 | p=NS |
| [C01181](http://www.genome.jp/dbget-bin/www_bget?cpd:C01181) | gamma-Butyrobetaine | 146.1176 | 11.66 | 41 | 39 | 33 | 30 | 35 | 11 | 13 | 15 | 14 | 13 | 13 | 11 | 14 | 18 | 12 | p=NS |
| [C00064](http://www.genome.jp/dbget-bin/www_bget?cpd:C00064) | Gln | 147.0779 | 15.49 | 14084 | 12634 | 11076 | 10258 | 10885 | 10268 | 10382 | 9720 | 12017 | 11761 | 10126 | 9131 | 9794 | 12030 | 10747 | p=NS |
| [C00025](http://www.genome.jp/dbget-bin/www_bget?cpd:C00025) | Glu | 148.0624 | 15.76 | 11195 | 10786 | 12493 | 12730 | 14729 | 11881 | 13721 | 10983 | 14395 | 12637 | 12540 | 14600 | 13050 | 11410 | 12239 | p=NS |
| [C00329](http://www.genome.jp/dbget-bin/www_bget?cpd:C00329) | Glucosamine | 180.0872 | 13.42 | 1.7 | N.D. | N.D. | N.D. | N.D. | 1.9 | 2.1 | N.D. | N.D. | 2.3 | 2.3 | 1.6 | N.D. | 2.1 | 2.4 | p=NS |
| [C00127](http://www.genome.jp/dbget-bin/www_bget?cpd:C00127) | Glutathione(ox) | 307.0852 | 17.64 | 306 | 335 | 357 | 325 | 328 | 274 | 403 | 384 | 291 | 313 | 283 | 300 | 292 | 361 | 388 | p=NS |
| [C00051](http://www.genome.jp/dbget-bin/www_bget?cpd:C00051) | Glutathione(red) | 308.0931 | 19.18 | 1027 | 1169 | 1203 | 1228 | 1360 | 1376 | 1002 | 974 | 1181 | 1294 | 1407 | 1294 | 1257 | 1148 | 1125 | p=NS |
| [C00037](http://www.genome.jp/dbget-bin/www_bget?cpd:C00037) | Gly | 76.0394 | 11.95 | 852 | 936 | 862 | 821 | 883 | 735 | 890 | 854 | 851 | 699 | 703 | 626 | 846 | 822 | 806 | p=NS |
| [C02037](http://www.genome.jp/dbget-bin/www_bget?cpd:C02037) | Gly-Gly | 133.0605 | 12.06 | 6.2 | 5.8 | 5.9 | 6.0 | 6.6 | 6.4 | 5.8 | 6.8 | 6.3 | 5.8 | 7.2 | 4.5 | 7.5 | 5.1 | 7.0 | p=NS |
| [C00670](http://www.genome.jp/dbget-bin/www_bget?cpd:C00670) | Glycerophosphorylcholine | 258.1098 | 31.79 | 139 | 289 | 139 | 192 | 152 | 190 | 498 | 406 | 403 | 335 | 299 | 253 | 470 | 325 | 267 | p=NS |
| [C00581](http://www.genome.jp/dbget-bin/www_bget?cpd:C00581) | Guanidinoacetate | 118.0601 | 11.86 | 11 | 11 | 8.0 | 7.4 | 7.0 | 5.6 | 6.3 | 6.6 | 6.9 | 10 | 5.9 | 6.6 | 7.7 | 8.5 | 6.8 | p=NS |
| [C00387](http://www.genome.jp/dbget-bin/www_bget?cpd:C00387) | Guanosine | 284.0989 | 18.06 | 9.7 | 5.4 | 6.2 | 7.7 | 7.7 | 9.7 | 3.4 | 6.0 | 6.0 | 7.1 | 8.1 | 6.8 | 5.4 | 6.5 | 8.1 | p=NS |
| [C00135](http://www.genome.jp/dbget-bin/www_bget?cpd:C00135) | His | 156.0772 | 10.48 | 461 | 549 | 360 | 293 | 331 | 489 | 511 | 460 | 561 | 520 | 577 | 556 | 508 | 571 | 602 | p=NS |
| [C00388](http://www.genome.jp/dbget-bin/www_bget?cpd:C00388) | Histamine | 112.0851 | 6.84 | 2.0 | 5.1 | 7.2 | 2.8 | 4.4 | 2.9 | 4.6 | 4.8 | 2.4 | 4.2 | 3.1 | 2.7 | 4.6 | 3.1 | 4.7 | p=NS |
| [C00263](http://www.genome.jp/dbget-bin/www_bget?cpd:C00263) | Homoserine | 120.0641 | 14.55 | 4.5 | 4.7 | 5.4 | 5.8 | 5.1 | 7.2 | 8.8 | 9.4 | 10 | 9.7 | 9.5 | 8.9 | 10 | 8.0 | 8.7 | p=NS |
| [C01015](http://www.genome.jp/dbget-bin/www_bget?cpd:C01015) | Hydroxyproline | 132.0640 | 17.44 | 59 | 73 | 80 | 83 | 77 | 83 | 87 | 117 | 80 | 76 | 102 | 76 | 84 | 79 | 85 | p=NS |
| [C00519](http://www.genome.jp/dbget-bin/www_bget?cpd:C00519) | Hypotaurine | 110.0260 | 26.66 | 2310 | 2327 | 1191 | 1200 | 1268 | 1183 | 1261 | 1256 | 1284 | 1095 | 1224 | 1301 | 1337 | 2176 | 1073 | p=NS |
| [C00262](http://www.genome.jp/dbget-bin/www_bget?cpd:C00262) | Hypoxanthine | 137.0455 | 15.91 | 179 | 118 | 125 | 129 | 146 | 182 | 52 | 64 | 99 | 126 | 143 | 132 | 123 | 106 | 141 | p=NS |
| [C00407](http://www.genome.jp/dbget-bin/www_bget?cpd:C00407) | Ile | 132.1004 | 14.79 | 44 | 43 | 58 | 43 | 62 | 47 | 44 | 42 | 43 | 51 | 35 | 48 | 36 | 29 | 42 | p=NS |
| [C00294](http://www.genome.jp/dbget-bin/www_bget?cpd:C00294) | Inosine | 269.0894 | 28.51 | 709 | 488 | 407 | 463 | 480 | 627 | 214 | 401 | 308 | 435 | 549 | 424 | 420 | 372 | 503 | p=NS |
| [C00123](http://www.genome.jp/dbget-bin/www_bget?cpd:C00123) | Leu | 132.1006 | 14.94 | 78 | 85 | 103 | 84 | 111 | 85 | 87 | 84 | 80 | 91 | 87 | 89 | 68 | 54 | 89 | p=NS |
| [C00047](http://www.genome.jp/dbget-bin/www_bget?cpd:C00047) | Lys | 147.1132 | 9.89 | 901 | 989 | 925 | 876 | 929 | 1176 | 1162 | 1320 | 1295 | 1042 | 1404 | 1107 | 1180 | 988 | 1516 | p=NS |
| [C01026](http://www.genome.jp/dbget-bin/www_bget?cpd:C01026) | N,N-Dimethylglycine | 104.0696 | 15.80 | 11 | 14 | 6.7 | 7.6 | 9.0 | 5.8 | 4.9 | 7.5 | 6.2 | 7.4 | 6.3 | 5.5 | 6.3 | 6.3 | 8.5 | p=NS |
| [C03793](http://www.genome.jp/dbget-bin/www_bget?cpd:C03793) | N6,N6,N6-Trimethyllysine | 189.1587 | 10.34 | 5.8 | 6.8 | 6.1 | 6.0 | 7.0 | 8.3 | 8.7 | 7.7 | 9.8 | 8.4 | 8.2 | 7.5 | 8.4 | 7.8 | 9.3 | p=NS |
| [C00153](http://www.genome.jp/dbget-bin/www_bget?cpd:C00153) | Nicotinamide | 123.0547 | 10.61 | 993 | 886 | 830 | 821 | 806 | 977 | 622 | 834 | 725 | 776 | 890 | 828 | 870 | 742 | 861 | p=NS |
| [C00547](http://www.genome.jp/dbget-bin/www_bget?cpd:C00547) | Noradrenaline | 170.0802 | 12.99 | 0.98 | 2.8 | 3.3 | 2.7 | 3.6 | 2.8 | 3.3 | 2.9 | 3.0 | 1.2 | 1.2 | 2.3 | 3.2 | 0.86 | 2.4 | p=NS |
| [C02571](http://www.genome.jp/dbget-bin/www_bget?cpd:C02571) | o-Acetylcarnitine | 204.1249 | 13.00 | 130 | 153 | 191 | 283 | 122 | 91 | 110 | 137 | 61 | 96 | 101 | 112 | 64 | 116 | 94 | p=NS |
| [C00077](http://www.genome.jp/dbget-bin/www_bget?cpd:C00077) | Ornithine | 133.0961 | 9.80 | 9.3 | 11 | 9.9 | 6.2 | 11 | 7.6 | 8.8 | 8.6 | 9.2 | 6.3 | 14 | 8.7 | 7.8 | 9.1 | 9.9 | p=NS |
| [C00079](http://www.genome.jp/dbget-bin/www_bget?cpd:C00079) | Phe | 166.0861 | 16.05 | 59 | 60 | 55 | 43 | 55 | 51 | 47 | 46 | 52 | 49 | 63 | 55 | 51 | 49 | 59 | p=NS |
| [C00588](http://www.genome.jp/dbget-bin/www_bget?cpd:C00588) | Phosphorylcholine | 184.0727 | 30.21 | 407 | 356 | 291 | 266 | 298 | 374 | 339 | 266 | 346 | 372 | 405 | 346 | 372 | 399 | 379 | p=NS |
| [C00408](http://www.genome.jp/dbget-bin/www_bget?cpd:C00408) | Pipecolate | 130.0855 | 14.87 | 3.7 | 3.8 | 4.3 | 3.8 | 5.0 | 3.4 | 4.2 | 3.8 | 3.8 | 4.4 | 4.3 | 4.3 | 5.1 | 4.0 | 4.7 | p=NS |
| [C10172](http://www.genome.jp/dbget-bin/www_bget?cpd:C10172) | Proline betaine | 144.1017 | 16.71 | 6.3 | 6.4 | 3.8 | 4.7 | 5.0 | N.D. | N.D. | N.D. | N.D. | N.D. | N.D. | N.D. | N.D. | N.D. | N.D. | p=NS |
| [C00134](http://www.genome.jp/dbget-bin/www_bget?cpd:C00134) | Putrescine(1,4-Butanediamine) | 89.1065 | 6.74 | 3.9 | 4.2 | 3.8 | 4.1 | 3.8 | 3.7 | 5.5 | 5.3 | 6.0 | 7.3 | 4.8 | 5.3 | 5.1 | 4.8 | 4.9 | p=NS |
| [C00647](http://www.genome.jp/dbget-bin/www_bget?cpd:C00647) | Pyridoxamine 5'-phosphate | 249.0650 | 15.13 | 39 | 36 | 39 | 38 | 41 | 35 | 37 | 36 | 39 | 37 | 36 | 37 | 36 | 37 | 38 | p=NS |
| [C03451](http://www.genome.jp/dbget-bin/www_bget?cpd:C03451) | S-Lactoylglutathione | 380.1121 | 19.98 | 8.0 | 8.6 | 7.2 | 8.6 | 7.6 | 6.6 | 8.1 | 8.8 | 7.0 | 7.5 | 6.7 | 7.8 | 6.9 | 7.6 | 6.9 | p=NS |
| [C00021](http://www.genome.jp/dbget-bin/www_bget?cpd:C00021) | SAH | 193.0699 | 12.56 | 2.7 | 2.1 | 3.1 | 3.0 | 3.4 | 4.0 | 2.0 | 2.0 | 2.8 | 3.0 | 3.4 | 3.1 | 3.1 | 2.6 | 3.5 | p=NS |
| [C00019](http://www.genome.jp/dbget-bin/www_bget?cpd:C00019) | SAM+ | 200.0774 | 10.22 | 46 | 44 | 45 | 43 | 47 | 42 | 45 | 43 | 50 | 48 | 44 | 46 | 45 | 44 | 43 | p=NS |
| [C00213](http://www.genome.jp/dbget-bin/www_bget?cpd:C00213) | Sarcosine | 90.0541 | 13.66 | 4.5 | 4.0 | 4.4 | 3.2 | N.D. | 2.9 | 3.2 | 2.9 | N.D. | N.D. | 3.3 | 3.2 | 3.8 | 3.3 | 3.1 | p=NS |
| [C00065](http://www.genome.jp/dbget-bin/www_bget?cpd:C00065) | Ser | 106.0491 | 14.40 | 540 | 532 | 475 | 415 | 550 | 778 | 949 | 1041 | 1018 | 848 | 842 | 776 | 1031 | 992 | 998 | p=NS |
| [C00315](http://www.genome.jp/dbget-bin/www_bget?cpd:C00315) | Spermidine | 146.1644 | 6.48 | 35 | 38 | 30 | 33 | 37 | 25 | 34 | 41 | 35 | 35 | 28 | 31 | 32 | 36 | 35 | p=NS |
| [C00750](http://www.genome.jp/dbget-bin/www_bget?cpd:C00750) | Spermine | 102.1145 | 6.39 | 7.1 | 6.5 | 6.0 | 6.9 | 5.5 | 4.7 | 9.1 | 12 | 11 | 9.2 | 6.7 | 7.8 | 5.0 | 8.2 | 8.5 | p=NS |
| [C00245](http://www.genome.jp/dbget-bin/www_bget?cpd:C00245) | Taurine | 126.0211 | 32.33 | 20760 | 18734 | 22106 | 20156 | 22856 | 19993 | 20231 | 17078 | 24339 | 20758 | 21307 | 19770 | 22382 | 21839 | 19197 | p=NS |
| [C00378](http://www.genome.jp/dbget-bin/www_bget?cpd:C00378) | Thiamine | 265.1130 | 9.51 | 1.3 | 1.3 | 1.2 | 1.6 | 2.0 | 1.7 | 0.80 | 0.93 | 1.2 | 1.1 | 1.4 | 1.2 | 1.4 | 1.0 | 1.6 | p=NS |
| [C01081](http://www.genome.jp/dbget-bin/www_bget?cpd:C01081) | Thiamine monophosphate | 345.0785 | 15.47 | 42 | 33 | 32 | 39 | 41 | 33 | 15 | 16 | 27 | 25 | 35 | 31 | 28 | 29 | 34 | p=NS |
| [C00188](http://www.genome.jp/dbget-bin/www_bget?cpd:C00188) | Thr | 120.0645 | 15.18 | 410 | 434 | 446 | 462 | 512 | 607 | 691 | 590 | 607 | 612 | 754 | 644 | 574 | 485 | 755 | p=NS |
| [C01104](http://www.genome.jp/dbget-bin/www_bget?cpd:C01104) | Trimethylamine N-oxide | 76.0750 | 9.47 | 1.8 | 2.0 | 3.3 | 3.3 | 2.0 | 1.5 | 0.79 | 2.0 | N.D. | 2.8 | 0.81 | N.D. | 0.85 | N.D. | 0.76 | p=NS |
| [C00086](http://www.genome.jp/dbget-bin/www_bget?cpd:C00086) | Urea | 61.0388 | 30.95 | 7877 | 7946 | 7049 | 7109 | 9064 | 7664 | 8059 | 6366 | 7116 | 32375 | 10149 | 10824 | 8733 | 6931 | 9754 | p=NS |
| [C00299](http://www.genome.jp/dbget-bin/www_bget?cpd:C00299) | Uridine | 245.0785 | 32.46 | 480 | 301 | 374 | 377 | 456 | 504 | 219 | 283 | 525 | 496 | 537 | 491 | 424 | 448 | 515 | p=NS |
| [C00183](http://www.genome.jp/dbget-bin/www_bget?cpd:C00183) | Val | 118.0853 | 14.50 | 121 | 122 | 142 | 130 | 155 | 137 | 131 | 120 | 116 | 140 | 133 | 172 | 119 | 84 | 139 | p=NS |
| [C00385](http://www.genome.jp/dbget-bin/www_bget?cpd:C00385) | Xanthine | 153.0442 | 28.41 | 48 | 45 | 35 | 33 | 49 | 63 | 18 | 29 | 41 | 51 | 70 | 35 | 45 | 34 | 42 | p=NS |

Other cationic metabolites in the cardiac tissues of mice fed on a normal chow (NC) (n=3), high fat diet (HF) (n=3) and high fat diet+linagliptin (HF+Lina)(n=3) groups.

Data were analyzed by the 2-tailed Student’s t-test.
