# Supplementary material for: Inhibition of dipeptidyl peptidase-4 ameliorates cardiac ischemia and systolic dysfunction by up-regulating the FGF-2/EGR-1 pathway
Source: PLoS One. 2017 Aug 3;12(8):e0182422. doi: 10.1371/journal.pone.0182422 (PMC5542565; doi:10.1371/journal.pone.0182422)
Supplement: S2 Table — Other anionis metabolites in the cardiac tissues of mice fed on a normal chow (NC) (n = 3), high fat diet (HF) (n = 3) and high fat diet+linagliptin (HF+Lina)(n = 3) groups. Data were analyzed by the 2-tailed Student’s t-test. (DOCX) [file pone.0182422.s006.docx]

**Table S2 Anionic metabolites**

| nmol/g (Cardiac tissue weight) | |  |  |  |  |  |  |  |  |  |  |  |  |  |  |  |  |  |  |
| --- | --- | --- | --- | --- | --- | --- | --- | --- | --- | --- | --- | --- | --- | --- | --- | --- | --- | --- | --- |
|  |  | Group | | **NC** | | | | | **HF** | | | | | **HF+Lina** | | | | | **HF vs HF+LIna (StudentT)** |
|  |  | Tube no. | | NC1 | NC2 | NC3 | NC4 | NC5 | HF1 | HF2 | HF3 | HF4 | HF5 | HF+Lina1 | HF+Lina2 | HF+Lina3 | HF+Lina4 | HF+Lina5 |  |
|  |  | Ave m/z | Ave CorMT |  |  |  |  |  |  |  |  |  |  |  |  |  |  |  |  |
| [C06369](http://www.genome.jp/dbget-bin/www_bget?cpd:C06369) | 2-Deoxyglucose 6-phosphate | 243.0288 | 15.57 | 3.9 | 4.3 | 10 | 10 | 13 | 36 | 41 | 24 | 39 | 47 | 28 | 34 | 39 | 46 | 35 | p=NS |
| [C05984](http://www.genome.jp/dbget-bin/www_bget?cpd:C05984) | 2-Hydroxybutyrate | 103.0416 | 16.17 | 28 | 16 | 13 | 5.1 | 12 | 8.5 | 7.0 | 4.4 | 8.4 | 6.1 | 6.7 | 8.4 | 4.6 | 5.2 | 6.2 | p=NS |
| [C02630](http://www.genome.jp/dbget-bin/www_bget?cpd:C02630) | 2-Hydroxyglutarate | 147.0307 | 11.69 | 22 | 22 | 18 | 15 | 18 | 22 | 24 | 20 | 22 | 25 | 29 | 27 | 22 | 24 | 22 | p=NS |
| [C00631](http://www.genome.jp/dbget-bin/www_bget?cpd:C00631) | 2PG | 184.9872 | 11.05 | 45 | 36 | 47 | 30 | 39 | 28 | 30 | 45 | 46 | 44 | 32 | 43 | 32 | 48 | 34 | p=NS |
| [C01089](http://www.genome.jp/dbget-bin/www_bget?cpd:C01089) | 3-Hydroxybutyrate | 103.0418 | 16.59 | 75 | 45 | 29 | 32 | 54 | 39 | 32 | 21 | 45 | 26 | 27 | 41 | 38 | 31 | 36 | p=NS |
| [C00197](http://www.genome.jp/dbget-bin/www_bget?cpd:C00197) | 3PG | 184.9873 | 10.97 | 286 | 231 | 284 | 242 | 240 | 175 | 249 | 298 | 317 | 287 | 226 | 271 | 221 | 296 | 217 | p=NS |
| [C01879](http://www.genome.jp/dbget-bin/www_bget?cpd:C01879) | 5-Oxoproline | 128.0369 | 16.65 | 65 | 60 | 62 | 64 | 60 | 55 | 51 | 51 | 57 | 64 | 51 | 50 | 59 | 69 | 62 | p=NS |
| [C00345](http://www.genome.jp/dbget-bin/www_bget?cpd:C00345) | 6-Phosphogluconate | 275.0182 | 12.30 | 16 | 35 | 18 | 15 | 15 | 20 | 13 | 15 | 20 | 18 | 17 | 22 | 35 | 20 | 24 | p=NS |
| [C00024](http://www.genome.jp/dbget-bin/www_bget?cpd:C00024) | Acetyl CoA | 403.5572 | 15.49 | 4.7 | 5.4 | 6.0 | 7.6 | 3.0 | 3.1 | 7.6 | 11 | 3.5 | 3.3 | 3.4 | 3.4 | 1.9 | 5.4 | 2.8 | p=NS |
| [C00054](http://www.genome.jp/dbget-bin/www_bget?cpd:C00054) | Adenosine 3',5'-diphosphate | 426.0228 | 12.90 | 42 | 28 | 48 | 37 | 39 | 43 | 37 | 35 | 44 | 38 | 41 | 62 | 41 | 57 | 54 | p=NS |
| [C03794](http://www.genome.jp/dbget-bin/www_bget?cpd:C03794) | Adenylosuccinate | 462.0673 | 12.71 | 235 | 177 | 161 | 198 | 249 | 160 | 107 | 107 | 198 | 182 | 120 | 220 | 258 | 177 | 194 | p=NS |
| [C00008](http://www.genome.jp/dbget-bin/www_bget?cpd:C00008) | ADP | 426.0233 | 14.72 | 661 | 670 | 678 | 655 | 635 | 562 | 691 | 739 | 750 | 707 | 582 | 609 | 624 | 701 | 587 | p=NS |
| [C00498](http://www.genome.jp/dbget-bin/www_bget?cpd:C00498) | ADP-glucose | 588.0767 | 19.41 | 6.7 | 6.9 | 8.3 | 6.3 | 8.2 | 5.1 | 6.2 | 4.9 | 7.4 | 6.0 | 7.5 | 7.3 | 7.1 | 6.4 | 5.6 | p=NS |
| [C00301](http://www.genome.jp/dbget-bin/www_bget?cpd:C00301) | ADP-ribose | 558.0642 | 18.90 | 38 | 54 | 44 | 41 | 42 | 43 | 38 | 45 | 37 | 38 | 33 | 38 | 45 | 42 | 34 | p=NS |
| [C00020](http://www.genome.jp/dbget-bin/www_bget?cpd:C00020) | AMP | 346.0572 | 16.91 | 1339 | 2755 | 2060 | 1987 | 1864 | 1379 | 3870 | 3378 | 2072 | 1887 | 1245 | 1614 | 2336 | 2074 | 1628 | p=NS |
| [C00002](http://www.genome.jp/dbget-bin/www_bget?cpd:C00002) | ATP | 505.9887 | 14.03 | 87 | 66 | 92 | 89 | 80 | 48 | 127 | 123 | 114 | 85 | 68 | 73 | 62 | 105 | 68 | p=NS |
| [C08261](http://www.genome.jp/dbget-bin/www_bget?cpd:C08261) | Azelate | 187.0991 | 13.71 | 2.8 | 2.9 | 4.1 | 4.9 | 3.7 | 3.4 | 2.4 | 2.7 | 3.2 | 3.5 | 3.4 | 3.3 | 3.5 | 3.5 | 3.0 | p=NS |
| [C00307](http://www.genome.jp/dbget-bin/www_bget?cpd:C00307) | CDP-choline | 547.1210 | 27.57 | 37 | 36 | 41 | 35 | 26 | 21 | 20 | 34 | 14 | 23 | 16 | 20 | 12 | 11 | 15 | p=NS |
| [C00417](http://www.genome.jp/dbget-bin/www_bget?cpd:C00417) | cis-Aconitate | 173.0099 | 10.01 | 2.4 | 2.5 | 2.7 | 3.2 | 2.1 | 2.5 | 2.5 | 3.8 | 1.5 | 1.8 | 2.2 | 1.5 | 1.5 | 2.2 | 1.4 | p=NS |
| [C00158](http://www.genome.jp/dbget-bin/www_bget?cpd:C00158) | Citrate | 191.0212 | 10.20 | 107 | 101 | 106 | 138 | 81 | 83 | 71 | 108 | 64 | 79 | 92 | 65 | 75 | 89 | 75 | p=NS |
| [C00055](http://www.genome.jp/dbget-bin/www_bget?cpd:C00055) | CMP | 322.0452 | 16.36 | 19 | 37 | 30 | 21 | 22 | 18 | 51 | 34 | 29 | 21 | 24 | 22 | 27 | 29 | 23 | p=NS |
| [C00128](http://www.genome.jp/dbget-bin/www_bget?cpd:C00128) | CMP-N-acetylneuraminate | 613.1404 | 19.91 | 14 | 13 | 12 | 11 | 12 | 13 | 15 | 18 | 16 | 15 | 14 | 14 | 14 | 15 | 13 | p=NS |
| [C00010](http://www.genome.jp/dbget-bin/www_bget?cpd:C00010) | CoA | 382.5518 | 15.01 | 67 | 95 | 75 | 84 | 87 | 38 | 114 | 115 | 88 | 66 | 39 | 51 | 74 | 80 | 44 | p=NS |
| [C00346](http://www.genome.jp/dbget-bin/www_bget?cpd:C00346) | Ethanolamine phosphate | 140.0127 | 20.47 | 389 | 324 | 323 | 342 | 289 | 272 | 313 | 335 | 296 | 328 | 265 | 253 | 276 | 310 | 270 | p=NS |
| [C00354](http://www.genome.jp/dbget-bin/www_bget?cpd:C00354) | F1,6P | 338.9898 | 12.34 | 517 | 300 | 296 | 441 | 295 | 181 | 554 | 676 | 626 | 705 | 346 | 473 | 201 | 612 | 426 | p=NS |
| [C00085](http://www.genome.jp/dbget-bin/www_bget?cpd:C00085) | F6P | 259.0238 | 15.83 | 668 | 595 | 290 | 424 | 304 | 250 | 419 | 507 | 404 | 597 | 590 | 483 | 208 | 562 | 905 | p=NS |
| [C00016](http://www.genome.jp/dbget-bin/www_bget?cpd:C00016) | FAD | 784.1499 | 20.98 | 77 | 67 | 68 | 74 | 69 | 67 | 75 | 74 | 70 | 71 | 67 | 70 | 71 | 74 | 71 | p=NS |
| [C00122](http://www.genome.jp/dbget-bin/www_bget?cpd:C00122) | Fumarate | 115.0053 | 10.26 | 358 | 210 | 251 | 277 | 363 | 279 | 162 | 149 | 300 | 254 | 209 | 313 | 310 | 234 | 268 | p=NS |
| [C00103](http://www.genome.jp/dbget-bin/www_bget?cpd:C00103) | G1P | 259.0236 | 15.61 | 206 | 283 | 97 | 127 | 106 | 98 | 305 | 422 | 148 | 198 | 170 | 158 | 78 | 181 | 268 | p=NS |
| [C00092](http://www.genome.jp/dbget-bin/www_bget?cpd:C00092) | G6P | 259.0240 | 15.95 | 2608 | 2481 | 1216 | 1638 | 1246 | 1009 | 1598 | 2084 | 1560 | 2302 | 2358 | 1920 | 859 | 2249 | 3711 | p=NS |
| [C00035](http://www.genome.jp/dbget-bin/www_bget?cpd:C00035) | GDP | 442.0180 | 14.90 | 24 | 27 | 30 | 27 | 29 | 25 | 40 | 30 | 37 | 30 | 31 | 25 | 29 | 33 | 28 | p=NS |
| [C00096](http://www.genome.jp/dbget-bin/www_bget?cpd:C00096) | GDP-mannose | 604.0712 | 19.51 | 6.4 | 7.2 | 4.7 | 6.3 | 6.3 | 6.3 | 7.7 | 6.3 | 7.3 | 7.0 | 7.5 | 6.5 | 7.7 | 4.1 | 6.7 | p=NS |
| [C00257](http://www.genome.jp/dbget-bin/www_bget?cpd:C00257) | Gluconate | 195.0524 | 20.02 | 8.2 | 7.6 | 10 | 8.3 | 7.8 | 7.7 | 7.2 | 7.2 | 7.7 | 11 | 8.3 | 12 | 8.6 | 7.6 | 8.2 | p=NS |
| [C00489](http://www.genome.jp/dbget-bin/www_bget?cpd:C00489) | Glutarate | 131.0366 | 11.71 | 3.1 | 2.4 | 2.9 | 2.7 | 2.7 | 2.2 | N.D. | 1.3 | 2.0 | 2.7 | 1.8 | 3.4 | 2.4 | 2.8 | 2.3 | p=NS |
| [C00093](http://www.genome.jp/dbget-bin/www_bget?cpd:C00093) | Glycerophosphate | 171.0083 | 13.65 | 2611 | 1291 | 2198 | 2301 | 2715 | 2515 | 1205 | 1268 | 2092 | 2130 | 2294 | 2752 | 1722 | 2527 | 2599 | p=NS |
| [C00144](http://www.genome.jp/dbget-bin/www_bget?cpd:C00144) | GMP | 362.0513 | 17.23 | 239 | 241 | 244 | 222 | 227 | 239 | 236 | 236 | 238 | 241 | 235 | 217 | 224 | 254 | 240 | p=NS |
| [C00044](http://www.genome.jp/dbget-bin/www_bget?cpd:C00044) | GTP | 521.9824 | 14.32 | 6.1 | 5.5 | 10 | 8.2 | 9.2 | N.D. | 18 | 11 | 11 | 7.9 | N.D. | N.D. | N.D. | 9.9 | 6.5 | p=NS |
| [C05582](http://www.genome.jp/dbget-bin/www_bget?cpd:C05582) | Homovanillate | 181.0509 | 19.64 | 14 | 12 | 9.2 | 8.2 | 13 | 6.4 | 4.8 | 4.4 | 6.5 | 9.8 | 7.2 | 9.8 | 7.1 | 9.0 | 12 | p=NS |
| [C00130](http://www.genome.jp/dbget-bin/www_bget?cpd:C00130) | IMP | 347.0413 | 16.33 | 4709 | 3270 | 4078 | 4109 | 4063 | 4453 | 1968 | 1962 | 3740 | 4044 | 4733 | 4027 | 3683 | 4210 | 4120 | p=NS |
| [C05123](http://www.genome.jp/dbget-bin/www_bget?cpd:C05123) | Isethionate | 124.9924 | 14.05 | 18 | 19 | 19 | 19 | 18 | 15 | 18 | 16 | 17 | 26 | 16 | 14 | 17 | 22 | 17 | p=NS |
| [C00311](http://www.genome.jp/dbget-bin/www_bget?cpd:C00311) | Isocitrate | 191.0213 | 9.97 | 6.9 | 21 | 6.1 | 7.5 | 4.5 | 11 | 24 | 34 | 5.4 | 10 | 14 | 5.0 | 16 | 6.6 | 10 | p=NS |
| [C00186](http://www.genome.jp/dbget-bin/www_bget?cpd:C00186) | Lactate | 89.0268 | 14.80 | 6783 | 6219 | 5206 | 5773 | 5484 | 7397 | 4147 | 7274 | 6662 | 8472 | 8475 | 6269 | 6751 | 6178 | 6017 | p=NS |
| [C00711](http://www.genome.jp/dbget-bin/www_bget?cpd:C00711) | Malate | 133.0161 | 10.71 | 611 | 402 | 426 | 497 | 616 | 477 | 322 | 296 | 519 | 471 | 403 | 532 | 568 | 430 | 485 | p=NS |
| [C01042](http://www.genome.jp/dbget-bin/www_bget?cpd:C01042) | N-Acetylaspartate | 174.0425 | 12.36 | 18 | 20 | 20 | 16 | 15 | 14 | 12 | 11 | 11 | 13 | 12 | 12 | 16 | 12 | 10 | p=NS |
| [C04501](http://www.genome.jp/dbget-bin/www_bget?cpd:C04501) | N-Acetylglucosamine 1-phosphate | 300.0489 | 16.50 | 6.5 | 5.5 | 5.3 | 6.1 | 4.1 | 6.3 | 5.4 | 5.2 | 5.3 | 5.7 | 5.7 | 8.1 | 5.5 | 5.6 | 5.5 | p=NS |
| [C00357](http://www.genome.jp/dbget-bin/www_bget?cpd:C00357) | N-Acetylglucosamine 6-phosphate | 300.0496 | 17.23 | 3.2 | 2.9 | 3.5 | 2.8 | 3.5 | 4.3 | 2.1 | 2.5 | 4.4 | 3.3 | 4.8 | 4.0 | 4.0 | 3.4 | 6.0 | p=NS |
| [C00003](http://www.genome.jp/dbget-bin/www_bget?cpd:C00003) | NAD+ | 662.1015 | 29.99 | 299 | 372 | 301 | 317 | 294 | 164 | 441 | 316 | 332 | 273 | 184 | 257 | 245 | 335 | 226 | p=NS |
| [C00004](http://www.genome.jp/dbget-bin/www_bget?cpd:C00004) | NADH | 664.1174 | 19.86 | 54 | 54 | 58 | 70 | 60 | 29 | 79 | 36 | 75 | 52 | 25 | 51 | 39 | 58 | 31 | p=NS |
| [C00006](http://www.genome.jp/dbget-bin/www_bget?cpd:C00006) | NADP+ | 742.0688 | 16.79 | 68 | 49 | 62 | 54 | 58 | 48 | 56 | 38 | 62 | 53 | 43 | 56 | 51 | 78 | 52 | p=NS |
| [C00005](http://www.genome.jp/dbget-bin/www_bget?cpd:C00005) | NADPH | 371.5393 | 14.35 | 20 | 12 | 27 | 25 | 20 | 9.3 | 23 | 7.0 | 29 | 10 | 9.4 | 19 | 9.7 | 20 | 11 | p=NS |
| [C00864](http://www.genome.jp/dbget-bin/www_bget?cpd:C00864) | Pantothenate | 218.1045 | 21.94 | 35 | 30 | 40 | 31 | 40 | 40 | 41 | 48 | 34 | 70 | 52 | 58 | 52 | 58 | 72 | p=NS |
| [C00074](http://www.genome.jp/dbget-bin/www_bget?cpd:C00074) | PEP | 166.9765 | 10.75 | 13 | 10 | 9.5 | 8.9 | 9.6 | 8.7 | 7.3 | 13 | 14 | 15 | 14 | 11 | 10 | 13 | 9.7 | p=NS |
| [C05682](http://www.genome.jp/dbget-bin/www_bget?cpd:C05682) | Phosphonoacetate | 138.9819 | 11.00 | 6.6 | 6.6 | 8.4 | N.D. | 6.1 | N.D. | 8.6 | 6.6 | 7.3 | N.D. | 8.4 | 8.5 | N.D. | N.D. | N.D. | p=NS |
| [C00199](http://www.genome.jp/dbget-bin/www_bget?cpd:C00199) | Ru5P | 229.0131 | 14.62 | 312 | 209 | 262 | 249 | 267 | 322 | 103 | 136 | 197 | 249 | 315 | 246 | 244 | 232 | 291 | p=NS |
| [C05382](http://www.genome.jp/dbget-bin/www_bget?cpd:C05382) | S7P | 289.0336 | 16.31 | 17 | 8.4 | 14 | 11 | 12 | 18 | 6.5 | 5.5 | 9.8 | 12 | 17 | 10 | 12 | 12 | 20 | p=NS |
| [C00042](http://www.genome.jp/dbget-bin/www_bget?cpd:C00042) | Succinate | 117.0206 | 10.77 | 296 | 309 | 278 | 312 | 362 | 284 | 326 | 260 | 284 | 292 | 215 | 365 | 289 | 274 | 293 | p=NS |
| [C06337](http://www.genome.jp/dbget-bin/www_bget?cpd:C06337) | Terephthalate | 165.0204 | 11.72 | 1.4 | 1.3 | 1.6 | 1.9 | 1.8 | 0.93 | 1.3 | 0.77 | 2.1 | 1.3 | 1.4 | 1.3 | 1.3 | 1.5 | 0.79 | p=NS |
| [C01620](http://www.genome.jp/dbget-bin/www_bget?cpd:C01620) | Threonate | 135.0317 | 17.14 | 93 | 84 | 101 | 98 | 95 | 85 | 108 | 79 | 97 | 107 | 89 | 94 | 87 | 105 | 96 | p=NS |
| [C00689](http://www.genome.jp/dbget-bin/www_bget?cpd:C00689) | Trehalose 6-phosphate | 421.0808 | 19.04 | 2.1 | 3.0 | N.D. | 2.5 | 2.4 | 2.7 | 5.2 | 3.3 | N.D. | N.D. | 3.3 | N.D. | 2.2 | N.D. | N.D. | p=NS |
| [C00015](http://www.genome.jp/dbget-bin/www_bget?cpd:C00015) | UDP | 402.9945 | 14.03 | 9.1 | 7.1 | 6.6 | 6.5 | 5.6 | 5.5 | 7.4 | 5.9 | 7.9 | 8.8 | 7.0 | 6.1 | 4.6 | 5.6 | 4.3 | p=NS |
| [C00029](http://www.genome.jp/dbget-bin/www_bget?cpd:C00029) | UDP-glucose | 565.0479 | 18.52 | 11 | 30 | 16 | 23 | 12 | 8.8 | 26 | 11 | 12 | 16 | 3.6 | 8.4 | 14 | 14 | N.D. | p=NS |
| [C00043](http://www.genome.jp/dbget-bin/www_bget?cpd:C00043) | UDP-N-acetylglucosamine | 606.0750 | 19.02 | 68 | 73 | 60 | 62 | 61 | 63 | 73 | 72 | 66 | 66 | 68 | 61 | 72 | 67 | 73 | p=NS |
| [C00105](http://www.genome.jp/dbget-bin/www_bget?cpd:C00105) | UMP | 323.0291 | 16.04 | 98 | 172 | 136 | 112 | 106 | 95 | 210 | 167 | 137 | 109 | 99 | 113 | 131 | 125 | 101 | p=NS |
| [C00366](http://www.genome.jp/dbget-bin/www_bget?cpd:C00366) | Urate | 167.0230 | 17.85 | 15 | 16 | 18 | 16 | 17 | 20 | 12 | 14 | 19 | 19 | 24 | 13 | 20 | 17 | 16 | p=NS |

Other anionis metabolites in the cardiac tissues of mice fed on a normal chow (NC) (n=3), high fat diet (HF) (n=3) and high fat diet+linagliptin (HF+Lina)(n=3) groups.

Data were analyzed by the 2-tailed Student’s t-test.
